# Resting-state functional MRI in treatment-resistant schizophrenia

**DOI:** 10.3389/fnimg.2023.1127508

**Published:** 2023-04-06

**Authors:** Noora Tuovinen, Alex Hofer

**Affiliations:** Division of Psychiatry I, Department of Psychiatry, Psychotherapy, Psychosomatics and Medical Psychology, Medical University of Innsbruck, Innsbruck, Austria

**Keywords:** schizophrenia, resting-state functional MRI, functional connectivity, treatment resistance, treatment response

## Abstract

**Background:**

Abnormalities in brain regions involved in the pathophysiology of schizophrenia (SCZ) may present insight into individual clinical symptoms. Specifically, functional connectivity irregularities may provide potential biomarkers for treatment response or treatment resistance, as such changes can occur before any structural changes are visible. We reviewed resting-state functional magnetic resonance imaging (rs-fMRI) findings from the last decade to provide an overview of the current knowledge on brain functional connectivity abnormalities and their associations to symptoms in treatment-resistant schizophrenia (TRS) and ultra-treatment-resistant schizophrenia (UTRS) and to look for support for the dysconnection hypothesis.

**Methods:**

PubMed database was searched for articles published in the last 10 years applying rs-fMRI in TRS patients, i.e., who had not responded to at least two adequate treatment trials with different antipsychotic drugs.

**Results:**

Eighteen articles were selected for this review involving 648 participants (TRS and control cohorts). The studies showed frontal hypoconnectivity before the initiation of treatment with CLZ or riluzole, an increase in frontal connectivity after riluzole treatment, fronto-temporal hypoconnectivity that may be specific for non-responders, widespread abnormal connectivity during mixed treatments, and ECT-induced effects on the limbic system.

**Conclusion:**

Probably due to the heterogeneity in the patient cohorts concerning antipsychotic treatment and other clinical variables (e.g., treatment response, lifetime antipsychotic drug exposure, duration of illness, treatment adherence), widespread abnormalities in connectivity were noted. However, irregularities in frontal brain regions, especially in the prefrontal cortex, were noted which are consistent with previous SCZ literature and the dysconnectivity hypothesis. There were major limitations, as most studies did not differentiate between TRS and UTRS (i.e., CLZ-resistant schizophrenia) and investigated heterogeneous cohorts treated with mixed treatments (with or without CLZ). This is critical as in different subtypes of the disorder an interplay between dopaminergic and glutamatergic pathways involving frontal, striatal, and hippocampal brain regions in separate ways is likely. Better definitions of TRS and UTRS are necessary in future longitudinal studies to correctly differentiate brain regions underlying the pathophysiology of SCZ, which could serve as potential functional biomarkers for treatment resistance.

## 1. Introduction

Following the consensus criteria of the Treatment Response and Resistance in Psychosis (TRRIP) working group, failure of at least two adequate treatment episodes with different antipsychotic drugs is required to establish treatment-resistant schizophrenia (TRS) (Howes et al., 2017). Approximately 30% of individuals diagnosed with schizophrenia (SCZ) do not respond to first-line antipsychotics and hence meet these criteria (Kane et al., 2019). Of note, they have poorer outcomes when compared to other individuals suffering from SCZ (Chakrabarti, 2021). Strong predictors for TRS include young age of onset, poor premorbid functioning, a higher severity of negative symptoms, a gradual mode of onset, a longer duration of untreated psychosis, and a higher number of relapses (Smart et al., 2021). Moreover, a varied percentage (10–60%) of individuals who show symptom improvement at the beginning of antipsychotic treatment develop TRS over time (Kane et al., 2019).

Clozapine (CLZ) is considered the only indicated and evidence-based treatment for TRS, and approximately 40% of people with TRS show symptom improvement when treated with this compound (Siskind et al., 2017). Resistance to treatment with CLZ constitutes a distinct, probably the most severely ill subgroup, namely CLZ-resistant or ultra-treatment-resistant SCZ (UTRS; Campana et al., 2021). In this subgroup of patients, augmentation of CLZ with electroconvulsive therapy (ECT) has been shown to be one of the most efficacious treatment strategies (Grover et al., 2022). During ECT, an electrical stimulus is used to elicit a generalized seizure in the anesthetized patient. Although effective, the exact mechanisms of ECT are unknown (Leaver et al., 2022). As an alternative to CLZ and ECT, riluzole which reduces brain glutamate levels may improve symptoms in TRS (Farokhnia et al., 2014). If clinical response to a single antipsychotic such as CLZ is inadequate, combinations of antipsychotics may also be used.

Differences in symptom presentation and treatment response make SCZ a heterogeneous disorder whose underlying pathophysiology is not clear. However, an interplay between dopaminergic and glutamatergic pathways involving frontal, striatal, and medial temporal lobe (particularly hippocampi) brain regions in separate ways in different subtypes of the disorder has been suggested (Howes et al., 2015). In fact, abnormalities in brain regions involved in the pathophysiology of SCZ may provide insight into individual clinical symptoms and treatment response. Knowledge of such regions may further help in diagnosing, monitoring, and developing specific treatment strategies for SCZ.

**Figure 1:**
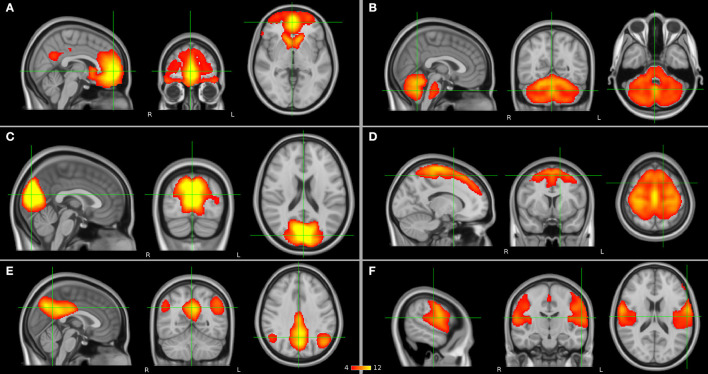
Resting-state networks (in orange) which have been suggested to have abnormalities in schizophrenia (SCZ) and as presented by independent component analysis (ICA): **(A)** frontal, **(B)** cerebellar, **(C)** visual, **(D)** somatomotor network (SMN), **(E)** default mode network (DMN), and **(F)** auditory networks.

To date, the diagnosis of SCZ relies on the use of diagnostic manuals in the lack of reliable structural brain abnormalities. As functional changes seem to precede structural changes in SCZ (Friston et al., 2016), it has for a long while been hypothesized that SCZ is a brain dysconnection syndrome (Stephan et al., 2009). Automatic diagnosis of SCZ based on brain functional connectivity is considered a promising tool (Algumaei et al., 2022). Furthermore, functional connectivity may provide more accurate prediction biomarkers and markers of response to different treatment strategies compared with structural information in SCZ. Communication between brain regions and networks can be studied with resting-state functional magnetic resonance imaging (rs-fMRI). The fMRI signal is based on a phenomenon called the blood-oxygen-level-dependent (BOLD) effect, which is an indirect measure of neural activity in which the electrical activity of neurons is coupled with the hemodynamic response function of the brain. Functional connectivity at rest, in the absence of a specific task, can be estimated through seed-based analysis or independent component analysis (ICA) approaches in which a statistical correlation analysis is applied between BOLD signal time courses of regions and networks in the brain. If brain regions have similar time courses, they are considered to be functionally connected and communicating with each other. In a seed-based analysis, an a priori region of interest (ROI) is selected, whereas the ICA is a data-driven method. As a result from these analyses, spatially distributed regions of the brain which present neuronal correlates of spontaneous fluctuations are identified as the so-called resting-state networks such as the frontal, the cerebellar, the visual, the somatomotor (SMN), the default mode (DMN), and the auditory networks (Figure 1) which may show abnormalities in the presence of SCZ. In addition to seed-based and ICA-based approaches, graph theoretical methods represent a powerful framework to study brain topology. They can be deployed to understand the dynamics of the functional networks and the architecture of the whole brain. By definition, a network can be modeled as a graph consisting of nodes (regions) linked through edges (connections between regions). Every edge represents the strength of coupling between the involved nodes. A network graph can be characterized by several nodal measures such as global and local efficiency as well as betweenness and degree centrality, which provide information on global and local brain connectivity. In this way, functional integration and segregation between different regions can be studied, and each region of the brain can be assigned a role in these mechanisms and important functional brain hubs can be identified (Sporns, 2013). In addition, a regional homogeneity (ReHo) analysis approach can be applied to depict local connectivity by looking at the synchronization between BOLD signals of a given voxel with its neighboring voxels (Jiang and Zuo, 2016).

Previous SCZ literature provided evidence for functional dysconnectivity (Fornito et al., 2012) and an atypical topology of important network hubs involving prefrontal, limbic, temporal, and parietal brain regions (Rubinov and Bullmore, 2013). Up to now, there is no clear consensus of the possible link of functional irregularities to pathophysiological mechanisms in SCZ. Functional connectivity increases and decreases may relate specifically to response differences to pharmacological treatment (e.g., first-line responders, initial and later onset TRS, UTRS) and this may explain some inconsistencies in the results. In fact, a previous review on treatment resistant and responding patients including rs-fMRI studies concluded that there are disruptions in areas involved in auditory and visual processing in both TRS and non-TRS (NTRS), that SMN changes appear in the context of TRS but not in NTRS, and that they are affected by treatment with CLZ (Chan et al., 2019). Another review reported alterations of activation and functional connectivity in fronto-temporal, corticostriatal, DMN, and salience networks, and of their interplay in TRS, but concluded that small sample sizes without adequate control cohorts limited the generalizability of the results (Molent et al., 2019). Identifying such irregularities in different SCZ subtypes represents a potential biomarker for treatment response or resistance. Although previous reviews gave support for functional differences in TRS, they had some limitations: next to rs-fMRI studies, they included studies which applied task-based fMRI and perfusion-based imaging. Further, they included rs-fMRI studies which were acquired with 1.5 Tesla (in addition to 3.0 Tesla) scanners and studies which provided no clear definition for TRS or UTRS. Therefore, the studies included may not have been well-comparable. In addition, new studies in ECT cohorts have emerged since these last reviews.

The aim of the present review is to summarize rs-fMRI findings (acquired with 3.0 Tesla MRI scanners) from the last decade to provide an overview of the current knowledge on brain functional connectivity abnormalities and their associations to symptoms in TRS and UTRS (in studies which applied the TRRIP consensus guidelines) and to look for support for the dysconnection hypothesis in SCZ.

## 2. Methods

### 2.1. Search strategy

PubMed database search was performed for articles published in the last 10 years (i.e., December 2012-November 2022) with the following terms in the title or in the abstract: schizophren^*^ OR psychosis AND functional connectivity OR rs-fMRI OR resting-state OR rs OR resting AND resistance OR resistant OR non-responsive OR TRS OR treatment resist^*^ OR treatment refract^*^ OR treatment-resistant refract^*^ OR treatment response OR response to treatment OR electroconvulsive therapy OR clozapine.

### 2.2. Study eligibility and selection

**Figure 2:**
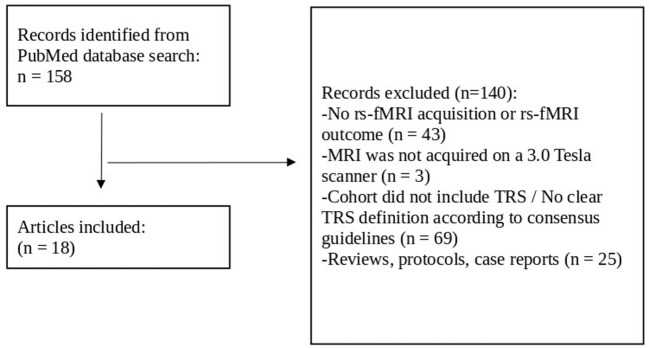
Flow chart review process for article selection. Rs-fMRI, resting-state functional magnetic resonance imaging; TRS, treatment-resistant schizophrenia.

Article titles and abstracts were screened by one of the authors (NT) from which articles were chosen for full-text review. They were assessed for inclusion in the review against the following eligibility criteria. Articles were included if the cohort investigated individuals with SCZ as confirmed either by the Mini-International Neuropsychiatric Interview (M.I.N.I.), or by the Structured Clinical Interview for DSM, or by fulfilling the criteria according to ICD-10. In addition, a failure of at least two adequate treatment episodes with different antipsychotic drugs according to TRRIP working group criteria for TRS was required (Howes et al., 2017). Articles were excluded if: (1) there were either no rs-fMRI acquisition or no rs-fMRI measures as an outcome, (2) the rs-fMRI was not acquired with a 3.0 Tesla MR scanner, (3) the cohort did not include TRS or TRS was not clearly defined as “not responding to at least two different antipsychotics despite adequate dosage and duration,” or (4) the article was a case report, a study protocol, or a review. Figure 2 reports the flow chart of the article selection process.

### 2.3. Data collection

The following data were recorded: (1) study characteristics (authors, year of publication, journal), (2) inclusion criteria (diagnostic criteria for SCZ, TRS definition), (3) participant cohorts (SCZ, control cohorts [e.g., healthy controls, NTRS], sample size, age, sex), (4) treatment, type and dosage of medication, (5) study design (cross-sectional, longitudinal), 6) MRI methodology (scanner field strength, MRI metrics [rs-fMRI sequence: length, parameters, sequence type (e.g., multiband), additional fieldmap sequence acquisition, eyes open / closed during rs-fMRI acquisition]), (7) functional connectivity analysis (preprocessing, analysis methods), (8) correlation analyses of clinical scores with connectivity, (9) main functional connectivity results, (10) correlations between connectivity and clinical variables, and prediction analysis findings.

## 3. Results

### 3.1. Study selection

Eighteen (out of 158) articles were selected for this review involving 648 participants (TRS and control cohorts) as presented in Table 1. Studies are presented by author and the year of publication, cohort(s), diagnostic criteria, TRS definition, experimental design (cross-sectional / longitudinal, treatment regime), and current antipsychotic treatment (chlorpromazine [CPZ] equivalents for TRS). Twelve of these studies were not included in previous reviews by Chan et al. (2019) and Molent et al. (2019).

**Table 1:** Characteristics of included studies for treatment-resistant schizophrenia (TRS) which applied resting-state functional magnetic resonance imaging (rs-fMRI).

**References**	**Cohort (*n*): Male/female, age years (SD)**	**Diagnostic criteria**	**TRS definition**	**Design**	**Current antipsychotic treatment, CPZ equivalents for TRS [mg (SD)]**
McNabb et al. (2018a)	TRS (15), M/F = 13/2, Age = 25.4 (5.1) NTRS (10), M/F = 10/4, Age = 29.1 (8.35)	DSM-IV diagnosis for SCZ	Have failed at least two 6-week trials with first-line antipsychotic drugs, present with persistent positive or negative symptoms contributing to a PANSS score ≥50 during screening	Cross-sectional before CLZ treatment	Ami = 1, Ari = 2, Ari + Ola = 2, Ola = 3, Ola + Que = 1, Pali = 3, Ris = 3 547.1 (263.7)
McNabb et al. (2018b)	TRS (18): M/F = 13/5, Age = 34.5 (15.4), NTRS (18): M/F = 14/4, Age = 30.0 (12.3), UTRS (16): M/F = 13/3, Age = 34.3 (10.8), HC (17): M/F = 15/2, Age = 32.7 (11.5)	DSM-IV diagnosis for SCZ	TRS: Had failed at least two previous (6–8 weeks) trials of atypical antipsychotics, were receiving CLZ. UTRS: Had failed at least two previous (6–8 weeks) trials of atypical antipsychotics and had also failed an adequate trial of CLZ monotherapy (at least 8 weeks post titration; Mouaffak et al., 2006)	Cross-sectional with TRS on CLZ treatment	In TRS: CLZ = 18 359.2 (275.5) In UTRS: CLZ + Ami = 5, Ris + Que = 1, CLZ + Ari = 4, Que + Ari = 2, CLZ + Que = 2, CLZ + Ris = 2 772.1 (522.6)
Ganella et al. (2017)	TRS (42): M/F = 30/13, Age = 41.3 (10.0), HC (42): M/F = 24/17, Age = 38.4 (10.4)	M.I.N.I. to confirm diagnosis of SCZ	At least two unsuccessful trials of two or more different antipsychotic types and currently taking CLZ (Kane et al., 1988; Suzuki et al., 2012)	Cross-sectional with TRS on CLZ treatment	CLZ = 42, other information not reported, 615.4 (55.84)
Ganella et al. (2018)	TRS (42): M/F = 30/13, Age = 41.3 (10.0), HC (42): M/F = 24/17, Age = 38.4 (10.4), UFM (16): M/F = 2/14, Age = 57.5 (11.7)	M.I.N.I. to confirm diagnosis of SCZ	At least two unsuccessful trials (4–10 weeks) of two or more different antipsychotic types (dosage equivalent to 1,000 mg/d CPZ) within the last 5 years, with a PANSS total score ≥90 and currently taking CLZ (Kane et al., 1988; Suzuki et al., 2012)	Cross-sectional with TRS on CLZ treatment	CLZ = 42, other information not reported, 615.4 (55.84)
Kim et al. (2022)	TRS (50): M/F =32/18, Age = 42.64 (9.79), HC (61): M/F = 29/32, Age = 39.89 (9.52)	DSM-IV diagnosis for SCZ	Failure to respond to at least two different antipsychotic medications administered in adequate doses (equivalent to ≥600 mg/day of CPZ) for at least 6 weeks, and persistence of clinically relevant positive or negative symptoms (at least one positive or negative symptom with a PANSS score ≥ 4)	Cross-sectional with (U)TRS on mixed treatments including CLZ	Rather high dosage 915.33 (411.41)
Gao et al. (2018)	TRS (17): M/F = 10/7, Age = 31.24 (9.40), NTRS (17): M/F = 9/8, Age = 36.82 (9.12), HC (29): M/F = 16/13, Age = 32.73 (7.61)	DSM-IV diagnosis for SCZ	Criteria of International Psychopharmacology Algorithm Project	Cross-sectional with (U)TRS on mixed treatments including CLZ	CLZ = 1, CLZ + Ris = 3, CLZ + Ari = 2, CLZ + Ola = 2, CLZ + Per = 1, Ola + Hal = 1, Ola + Per = 5, Que + Per = 1, Per = 1 696.47 (208.92)
White et al. (2016)	TRS (16): M/F = 12/4, Age = 36.69 (7.86), NTRS (22): M/F = 19/3, Age = 37.55 (9.60), HC (20): M/F = 17/3, Age = 36.30 (9.38)	DSM-IV diagnosis for SCZ	Modified Kane criteria for TRS on the basis of: completion of at least two sequential 4-week antipsychotic trials at a daily dose of 400–600 mg CPZ (or equivalent); persistent psychotic symptoms of at least moderate severity (as indexed by PANSS scores on one or more positive subscale measure); and impaired occupational functioning [as indexed by a score ≤ 59 on the GAF scale (Conley and Kelly, 2001; Demjaha et al., 2012)]	Cross-sectional with TRS on mixed treatments including CLZ	CLZ = 11, Ari = 2, Ola = 2, Ami = 1, Hal = 1, Pali = 1 Que = 1, Zuclopenthixol = 1 764.06 (339.15)
Alonso-Solís et al. (2015)	AVH (19): M/F = 13/6, Age = 40.05 (8.9), NAVH (14): M/F = 8/6, Age =36.43 (7.1), HC (20): M/F = 13/7, Age =37.75 (7.4)	DSM-IV- TR (Textrevision)	Medication-resistant AVH defined as daily presence of AVH in the past year, in face of at least two adequate trials of antipsychotic drugs at equivalent doses to 600 mg/day of CLZ	Cross-sectional with (U)TRS on mixed treatments including CLZ	Atypical Antipsychotics (i.e., Pali, Zip, CLZ, Ami, Que, Ris, Ari, Ola) = 15, Combination of one typical and one atypical antipsychotic = 4, Biperiden and trihexfenidil = 3, Benzodiazepines = 9, SSRI or SNRI = 5 CPZ not reported
Blazer et al. (2022)	TRS (21^**^): M/F = 16/6, Age = 35.0 (9.2)	SCID-I (IV)	Guided by recommendations of the TRRIP working group; exhibited chronic psychotic symptoms with a score of least a 4 (moderate) on one or more of the BPRS psychosis measures (hallucinatory behavior, unusual thought content, or conceptual disorganization); had at least two failed trials of non-CLZ antipsychotic drugs for at least 6 weeks; and no CLZ for at least 4 weeks if prior CLZ treatment occurred	Longitudinal: before CLZ initiation and 12 weeks thereafter	Baseline equivalent dose of non-CLZ antipsychotic drugs = 550.65 (479.73) Baseline CLZ dose = 69.79 (58.39) Follow-up equivalent dose of non-CLZ antipsychotic drugs = 288.61 (371.64) Follow-up CLZ dose = 337.50 (119.21)
Sarpal et al. (2022a)	TRS (18^*^): M/F = 13/6, Age = 36.0 (11.1)	SCID-I (IV)	Guided by the TRRIP working group consensus criteria: psychotic symptoms determined by a score of least a 4 on one or more of the BPRS psychosis measures; at least two failed trials of non-CLZ antipsychotic drugs for documented periods of at least 6 weeks; no CLZ for at least 4 weeks if prior CLZ treatment occurred	Longitudinal: before CLZ initiation and 12 weeks thereafter	Baseline Equivalent dose of non-CLZ antipsychotic drugs = 398.11 (40.3) CLZ dose at follow-up = 352.6 (109.6)
Pillinger et al. (2019)	TRS (19): M/F = 16/3, Age = 39.68 (10.92), HC (17^***):^ M/F = 15/3, Age = 36.28 (9.17)	DSM-IV diagnosis for SCZ	Presence of at least one positive and one negative symptom rated ≥ 4 on the PANSS, indicative of at least moderate severity, and a score of < 60 on the GAF scale, indicative of at least moderate functional impairment, despite 2 trials of an antipsychotic	Longitudinal: Pre- and post-riluzole treatment	Long-acting injectable antipsychotic medication = 9, Ris = 5, zuclopenthixol decanoate = 1, Ari = 2, Pali = 5, Ola = 5, Ami = 3, Que = 1, dual antipsychotic treatment = 2 CPZ not reported
Huang et al. (2018), Jiang et al. (2019a,b), Wang et al. (2020), and Hu Q. et al. (2022)	MSZ (21): M/F = 10/11, Age = 29.2 (7.1), DSZ (21): M/F = 9/12, Age = 30.7 (7.8), HC (23): M/F = 11/12, Age = 31.2 (5.9)	SCID-I/P (IV-TR, Patient edition)	Had not responded to two or more adequate antipsychotic trials in the past 5 years	Longitudinal: Before modified ECT and 4 weeks post-ECT	In MSZ: Ris + Ola = 3, Que + Pali ER = 1, Ami + Ari + CLZ = 1, CLZ + Ari = 1, Pali ER + Hal + Que = 1, Ris + Que = 1, Ris + Pali ER = 1, Pali ER + CLZ + Ami = 1, Ola + Hal = 1, Ola + per = 1, CPZ + Pali ER = 1, Zip + Ola = 1, Zip + Que + Ami = 1, Ola + Zip + CLZ = 1, Ola + Ami + Ris = 1, Ris = 2, Ola + Pali ER = 1, Pali ER = 1 604.6 (565.6)
Hu H. et al. (2022)	MSZ (21): M/F = 10/11, Age = 29.2 (7.1), DSZ (21^****^): M/F = 9/12, Age = 30.7 (6.9), HC (23): M/F = 11/12, Age = 31.2 (5.9)	SCID-I/P (IV-TR, Patient edition)	PANSS total score > 60	Longitudinal: Before modified ECT and 4 weeks post-ECT	Ris + Ola = 3, Que + Pali ER = 1, Ami + Ari + CLZ = 1, CLZ + Ari = 1, Pali ER + Hal + Que = 1, Ris + Que = 1, Ris + Pali ER = 1, Pali ER + CLZ + Ami = 1, Ola + Hal = 1, Ola + per = 1, CPZ + Pali ER = 1, Zip + Ola = 1, Zip + Que + Ami = 1, Ola + Zip + CLZ = 1, Ola + Ami + Ris = 1, Ris = 2, Ola + Pali ER = 1, Pali ER = 1 604.6 (565.6)
Yang et al. (2020)	TRS (47): M/F = 31/16, Age = 30.23 (9.65)	ICD-10	Treated with ECT under two conditions: had received at least two drugs in the acute phase (4 - 6 weeks each) but had shown no significant improvement, and assessed to have a very serious condition, such as suicidal individuals, who need rapid symptom control	Longitudinal: Before ECT and 4 weeks post-ECT	Information not available (supplementary material of the article not readable)

* rs-fMRI imaging on N = 21, but participant characteristics based on N = 22.

** rs-fMRI imaging on N = 18, but participant characteristics based on N = 19.

*** rs-fMRI imaging on N = 17, but participant characteristics based on N = 18.

**** Here the age SD for DSZ varies from what is reported for the other studies by the same research group (the original authors have confirmed that this is a typo).

Ami, Amisulpride; Ari, Aripiprazole; AVH, SCZ individuals with treatment-resistant auditory verbal hallucinations; BPRS, Brief Psychiatric Rating Scale (Hedlund and Vieweg, 1980); CLZ, clozapine; CPZ, chlorpromazine; DSM, The Diagnostic and Statistical Manual of Mental Disorders; DSZ, SCZ individuals taking only antipsychotics; ECT, electroconvulsive therapy; ER, extended-release; F, female; GAF, Global Assessment of Functioning (Hall and Parks, 1995); Hal, Haloperidol; HC, healthy controls; ICD, International Classification of Diseases; M, male; M.I.N.I., the Mini-International Neuropsychiatric Interview; MSZ, TRS individuals receiving a regular course of modified ECT combined with antipsychotics; NAVH, non-hallucinating SCZ individuals; NTRS, non-treatment-resistant SCZ; Ola, Olanzapine; Pali, Paliperidone; PANSS, Positive and Negative Syndrome Scale (Kay et al., 1987); Per, Perphenazine; Que, Quetiapine; Ris, Risperidone; rs-fMRI, resting-state functional magnetic resonance imaging; SCID-I, Structured Clinical Interview for DSM for Axis I Disorders; SCZ, schizophrenia; SD, standard deviation; SNRI, selective serotonin-noradrenaline reuptake inhibitors; SSRI, selective serotonin reuptake inhibitors; TRS, treatment-resistant SCZ; TRRIP, Treatment Response and Resistance in Psychosis (Howes et al., 2017); UFM, unaffected family members; UTRS, ultra-treatment-resistant SCZ; Zip, Ziprasidone.

### 3.2. Participant characteristics

Some of the studies presented patient cohort overlaps but applied different analysis methods. The cross-sectional CLZ study by Ganella et al. (2017) described with the same TRS and HC cohorts as Ganella et al. (2018) where an additional cohort of 16 unaffected family members (UFM) was investigated. Blazer et al. (2022) presented the data of three additional participants compared to Sarpal et al. (2022a) in their longitudinal CLZ study. The study on ECT by Huang et al. (2018), Jiang et al. (2019a,b), Wang et al. (2020), Hu H. et al. (2022), and Hu Q. et al. (2022) reported on exactly the same patient cohort of 21 TRS individuals receiving a regular course of modified ECT combined with antipsychotics (MSZ), 21 SCZ individuals taking only antipsychotics (DSZ), and 23 HC, although one of the mentioned studies (Hu H. et al., 2022) listed (likely by mistake) different age characteristics for DSZ.

### 3.3. Study designs

Eight of the studies were cross-sectional [i.e., in individuals who were CLZ-eligible (*n* = 1), currently on CLZ treatment (*n* = 3, two patient cohorts), currently on mixed treatments including CLZ (*n* = 4)], and the other ten studies were longitudinal from which two studies had a 12-week before and after CLZ treatment design (same patient cohort), one study applied rs-fMRI pre- and post-riluzole treatment, and seven studies had a 4-week pre- and post-ECT design (two patient cohorts).

### 3.4. Rs-fMRI sequence designs

A common length for the rs-fMRI sequence was between 7 and 10 minutes. There were, however, variations and the shortest rs-fMRI sequence lengths were only 5 min (McNabb et al., 2018a; Kim et al., 2022). The study by McNabb and coworkers applied a multiband fMRI acquisition with an additional fieldmap sequence (McNabb et al., 2018a). There were other studies which acquired longer rs-fMRI sequences (totaling 11.5 min over two separate acquisitions) with a multiband sequence (Blazer et al., 2022; Sarpal et al., 2022a). Multiecho fMRI sequence was also reported (Pillinger et al., 2019).

During rs-fMRI acquisition, some studies told the participants to keep their eyes open and to look at a fixation cross (White et al., 2016; McNabb et al., 2018a,b; Pillinger et al., 2019; Blazer et al., 2022; Sarpal et al., 2022a), while in other studies, they were asked to close their eyes (Alonso-Solís et al., 2015; Huang et al., 2018; Jiang et al., 2019a,b; Wang et al., 2020; Hu H. et al., 2022; Hu Q. et al., 2022; Kim et al., 2022). Some studies did not report whether the acquisitions were done with open or closed eyes (Ganella et al., 2017, 2018; Gao et al., 2018; Yang et al., 2020).

### 3.5. Data analysis methods

Data analysis methods, major significant functional connectivity results and their correlations to clinical symptoms, as well as prediction findings are displayed in Table 2. The studies reviewed applied ICA (*n* = 1), seed-based (*n* = 10), ReHo (*n* = 1), graph theory based (*n* = 4), graph-based global functional connectivity density (*n* = 1), and graph-based approaches for prediction (*n* = 1) analyses. Seed-based studies applied as seeds the striatum, the prefrontal cortex (PFC), the thalamus, cortical networks, the posterior cingulate cortex (PCC), the temporal parietal junction, the temporal region, the posterior inferior parietal lobule, the retrosplenial cortex, the hippocampus, the basal forebrain, the insula, and the anterior cingulate cortex (ACC) ROIs. Due to the unavailability of supplementary material for the study by Yang et al. (2020), it was not clear which were the 23 seed ROIs that have been used in their analysis. However, from their results it was possible to collect information for some of the regions studied (i.e., orbital PFC, superior and mediotemporal lobe, insula, hippocampus, parahippocampal gyrus, amygdala). Whole brain graph theory based (n=4) analyses used the automated anatomical labeling (AAL; Tzourio-Mazoyer et al., 2002) atlas for the main analyses.

**Table 2:** Major findings of TRS studies with rs-fMRI.

**References**	**Analysis methods**	**Functional connectivity findings**	**Clinical correlate and prediction findings**
McNabb et al. (2018a)	ICA	**CLZ-eligible** **>** **NTRS:** within the SMN (precuneus)	–
McNabb et al. (2018b)	Graph theory (116 region AAL atlas, custom-made atlas of 272 regions from the Human Brainnetome [Desikan-Killiany atlas] and the probabilistic MR atlas of the human cerebellum)	**UTRS** **<** **HC**: in three sub-networks (cerebellar-frontal, cingulo-frontal-temporal, frontoparietal)	No association.
Ganella et al. (2017)	Graph theory (116 region AAL atlas, Craddock atlas), local and global efficiency, strength of the network	**TRS on CLZ treatment** **<** **HC**: between fronto-temporal, fronto-occipital, temporo-occipital, and temporo-temporal lobes. The majority of reduced temporal lobe connections were located between Heschl's gyrus and the frontal lobe. The majority of reduced occipital lobe connections were located between the cuneus and the frontal lobe. The majority of reduced frontal lobe connections were located between the paracentral lobule and the occipital lobe. **Graph measures: TRS on CLZ treatment** **<** **HC:** Global efficiency **TRS on CLZ treatment** **>** **HC**: Local efficiency	No association.
Ganella et al. (2018)	Graph theory (116 region AAL atlas, 360 region Glasser atlas)	**TRS on CLZ treatment** **<** **HC** **>** **UFM:** predominantly in temporal (fusiform gyri) and occipital regions **TRS on CLZ treatment** **<** **HC, and HC** **=** **UFM**: predominantly in frontal (paracentral lobule and rolandic operculum) and temporal regions (Heschl's gyri) **Graph measures: TRS on CLZ treatment** **<** **HC:** Global efficiency **TRS on CLZ treatment** **>** **HC:** Local efficiency	No association.
Kim et al. (2022)	Seed-based [18 ROIs = 9 thalamus regions and 9 cortical networks (DMN, cingulo-opercular occipital, SMN, frontal parietal, lateral occipital, medial occipital, mediotemporal, temporal, and superior frontoparietal networks; Hwang et al., 2017)}, ROI-to-ROI, ROI-to-voxels	**Between thalamus ROIs and networks: TRS** **<** **HC:** between thalamic region 1 and thalamic region 2 as well as thalamic region 9; between thalamic region 2 and thalamic region 3 as well as thalamic region 4; between the frontoparietal and the mediotemporal as well as the superior frontoparietal networks; between the DMN and the cingulo-opercular occipital network; between the medial occipital and mediotemporal networks **TRS** **>** **HC:** between thalamic region 2 and the medial occipital network; between the cingulo-opercular occipital network and the medial occipital as well as the superior mediotemporal networks; between the mediotemporal network and the superior frontoparietal network **ROI to voxels connectivity: TRS** **<** **HC:** between thalamic region 3 ROI and the left intracalcarine cortex **TRS** **>** **HC:** between thalamic region 1 ROI and the left lingual gyrus; between thalamic 2 ROI and the left preCG; between thalamic region 3 ROI and the right supplementary motor cortex; between thalamic region 6 ROI and the frontal medial cortex, the left post-CG as well as the right preCG; between thalamic region 9 ROI and the left preCG	**TRS:** Connectivity between the frontoparietal and mediotemporal network was negatively correlated with positive, negative, and general symptoms, and PANSS total (sub)scores. Connectivity between the DMN and the mediotemporal network was negatively correlated with negative and general symptoms, and the PANSS total (sub)scores. Connectivity between thalamic region 3 and the right lingual gyrus was negatively correlated with positive, negative, and general symptoms, and the PANSS total (sub)scores. Connectivity between thalamic region 2 and the left preCG was positively correlated with general symptoms subscore of the PANSS.
Gao et al. (2018)	ReHo, classification (whether ReHo values differentiate between TRS, NTRS or HC)	**ReHo values TRS on mixed treatments** **<** **NTRS**: in the right angular gyrus **TRS on mixed treatments** **>** **NTRS:** in the left post-CG **TRS on mixed treatments** **<** **HC:** in the right fusiform gyrus, bilateral middle occipital gyri/middle temporal gyri, the right superior occipital gyrus, and the right superior parietal lobule **TRS on mixed treatments** **>** **HC:** in the right middle frontal gyrus/orbital part, the right putamen, bilateral inferior frontal gyri/triangular part, and bilateral superior medial frontal gyri	No association **Prediction:** The ReHo values in the left post-CG correctly classified 16 of 17 patients with TRS and 14 of 17 patients with NTRS. The optimal sensitivity of for differentiating TRS from NTRS was 94.12%, and the optimal specificity was 82.35%. The optimal sensitivity and specificity of the ReHo values in the left inferior frontal gyrus for differentiating TRS from HC were 100% (17/17) and 86.21% (25/29), respectively.
White et al. (2016)	Seed-based (four striatal ROIs = dorsal caudate, ventral striatum/nucleus accumbens, dorsocaudal putamen, ventral-rostral putamen; Dandash et al., 2014), ROI-to-voxels; prediction analysis (whether current PANSS subscores, or antipsychotic medication dosage predict striatal connectivity in TRS and NTRS)	**TRS on mixed treatments** **<** **HC:** between the dorsal caudate ROI and the sensorimotor cortex; between the ventral striatum ROI and the middle frontal gyrus; between the ventral-rostral putamen ROI and the striatum **TRS on mixed treatments** **<** **NTRS:** between the ventral striatum ROI and the substantia nigra; between the dorsocaudal putamen ROI and the pulvinar of the thalamus **TRS on mixed treatments** **>** **NTRS:** between the dorsal caudate ROI and the medial and superior PFC	**TRS on mixed treatments:** Reduced connectivities between the ventral striatum ROI and the PCC, the precuneus, as well as the middle frontal gyrus were associated with higher PANSS positive subscores. Increased connectivities between the dorsal striatum ROIs and the precuneus, the PCC, the medial PFC, the middle temporal gyrus, as well as the inferior and superior parietal lobules were associated with higher PANSS positive subscores. **Prediction:** In TRS, CPZ dosage positively predicted connectivities between the dorsal caudate and the lingual gyrus, the cerebellum, the fusiform gyrus, as well as the occipital lobe; between the dorsocaudal putamen and the lingual gyrus as well as the cuneus; and between the ventral-rostral putamen and the medial frontal gyrus. CPZ dosage negatively predicted connectivities between the ventral striatum and the PCC, the lingual gyrus, the middle frontal gyrus, as well as the cerebellum; and between the dorsal caudate and the post-CG.
Arango et al. (2003)	Seed-based (eleven DMN ROIs = PCC, anteromedial PFC, dorsomedial PFC, temporal parietal junction, lateral temporal cortex, temporal pole, ventromedial PFC, posterior inferior parietal lobule, retrosplenial cortex, parahippocampal cortex, hippocampal formation; Andrews-Hanna et al., 2010), ROI-to-voxels	**AVH (on mixed treatments)** **<** **HC, and AVH** **<** **NAVH:** between the ventromedial PFC ROI and bilateral paracingulate cortices, bilateral anterior cingulate cortices, as well as bilateral subcallosal cortices; between the hippocampal formation ROI and bilateral PCC as well as bilateral precunei **AVH** **>** **HC, and AVH** **>** **NAVH:** between the dorsomedial PFC ROI and bilateral central opercular cortices, bilateral insular cortices, bilateral preCG, as well as bilateral superior temporal gyri; between the temporal pole ROI and the cerebellum **AVH** **>** **HC, and NAVH** **>** **HC:** between the posterior inferior parietal lobule ROI and bilateral occipital fusiform gyri, bilateral lingual gyri, as well as the left occipital pole; between the retrosplenial cortex ROI and bilateral occipital cortices, bilateral intracalcarine cortices, the left occipital fusiform gyrus, as well as bilateral lingual gyri	–
Blazer et al. (2022)	Seed-based (seven ROIs = bilateral dorsal caudate, nucleus accumbens, bilateral ventral caudate, bilateral ventral rostral putamen; Di Martino et al., 2008), ROI-to-voxels; connectivity of networks (DMN, frontoparietal network, and the salience networks) with the striatum; prediction analysis (whether connectivity before initiation of CLZ treatment predicts antipsychotic efficacy)	**ROI analysis: Post-CLZ treatment** **>** **before initiation of CLZ treatment:** between the right dorsal caudate ROI and the right anterior insula as well as the right inferior frontal lobe **Network to ROI analysis: Post-CLZ treatment** **>** **before initiation of CLZ treatment:** between the frontoparietal network and the right dorsal caudate	Increased connectivities between the right dorsal caudate and the right anterior insula, the right inferior frontal lobe, as well as the frontoparietal network associated with a higher percent reduction in psychotic symptoms of the BPRS after 12-week CLZ treatment **Prediction:** Corticostriatal connectivities, i.e., between the right dorsal caudate and the right anterior insula as well as the right inferior frontal gyrus, before initiation of CLZ treatment predicted CLZ efficacy (reduction in positive symptoms).
Sarpal et al. (2022a)	Seed-based [three ROIs = bilateral cholinergic basal forebrain based on Eickhoff-Zilles atlas (Eickhoff et al., 2005; Zaborszky et al., 2008), the bilateral dorsolateral PFC (Sarpal et al., 2022b), bilateral anterior hippocampus (Neurosynth; https://neurosynth.org/), ROI-to-voxels]	–	Connectivity before initiation of CLZ treatment between the basal forebrain and the dorsolateral PFC was negatively correlated with the BPRS psychosis measure score. Connectivity change between the basal forebrain and the dorsolateral PFC was positively correlated with CLZ/n-desmethylclozapine ratio.
Pillinger et al. (2019)	Seed-based (Six ACC ROIs; Margulies et al., 2007), ROI-to-voxels	**Pre-riluzole TRS** **<** **HC:** between the ACC ROI and the right anterior PFC **Post-riluzole TRS** **>** **HC:** between the ACC ROI and the right anterior PFC	**Pre-riluzole TRS:** Lower functional connectivity between the ACC and the anterior PFC was associated with lower verbal learning scores of the AVLT
Hu Q. et al. (2022)	Seed-based [six ROIs = PCC (Buckner et al., 2008), medial PFC, bilateral angular gyri, bilateral middle temporal gyri (Groppe et al., 2011; Liao et al., 2011; Luo et al., 2011)], ROI-to-ROI	**MSZ pre-ECT** **>** **HC:** between the left angular gyrus and the right middle temporal gyrus **MSZ post-ECT** **>** **HC:** between the right angular gyrus and the left middle temporal gyrus; between the left angular gyrus and the right middle temporal gyrus **MSZ post-ECT** **>** **DSZ (treatment as usual) followup:** between the left angular gyrus and the right middle temporal gyrus; between the right angular gyrus and the left middle temporal gyrus **MSZ post-ECT** **>** **MSZ pre-ECT:** between the left angular gyrus and the right middle temporal gyrus; between the right angular gyrus and the left middle temporal gyrus	**MSZ:** The connectivity change between the right angular gyrus and the right middle temporal gyrus was positively associated with a reduction of the PANSS negative subscore. The connectivity change between the right angular gyrus and the right middle temporal gyrus was positively correlated with the post-treatment reduction ratio of the PANSS total score. The connectivity change between the left angular gyrus and the right middle temporal gyrus was positively correlated with the reduction of the PANSS general symptoms subscore.
Hu H. et al. (2022)	Graph theory (116 region AAL atlas), topological properties of brain networks (i.e., global efficiency, local efficiency, clustering coefficient, small-worldness), node metrics (i.e., degree, efficiency, betweenness centrality)	**MSZ pre-ECT versus post-ECT:** 22 brain regions change in at least one of the nodal measure (i.e., efficiency, betweenness centrality, degree), mainly located in the frontal lobe and cerebellum. **MSZ post-ECT** **>** **MSZ pre-ECT:** between several DMN regions and cerebellar regions, Local efficiency **MSZ post-ECT** **<** **MSZ pre-ECT:** Global efficiency	**MSZ:** The connectivity change between the left middle temporal gyrus and left cerebellar crus region 2 was correlated with the post-treatment reduction ratios of the PANSS total score and the PANSS general symptoms subscore. The connectivity change between the left inferior temporal gyrus and left cerebellar crus region 2 was correlated with the general symptoms reduction ratio of the PANSS. The connectivity change between the right angular gyrus and left cerebellar region 45 was correlated with the reductive ratio of the PANSS general psychopathology.
Wang et al. (2020)	Seed-based (16 thalamic ROIs; Fan et al., 2016), ROI-to-voxels	**MSZ post-ECT** **>** **pre-ECT:** between the right sensory thalamus ROI and the right putamen **MSZ post-ECT** **<** **pre-ECT:** between the left rostral temporal thalamus ROI and the left superior occipital cortex; between the left caudal temporal thalamus ROI and the left middle frontal cortex; between the right caudal temporal thalamus ROI and the left superior occipital cortex **MSZ** **>** **HC:** between the left rostral temporal thalamus ROI and the left superior occipital cortex; between the right caudal temporal thalamus ROI and the left superior occipital cortex **Refractory MSZ post-ECT** **>** **pre-ECT:** between the right posterior parietal thalamus ROI and the right inferior temporal cortex, as well as right cerebellar region 6 **Non-refractory MSZ post-ECT** **<** **pre-ECT:** between the right posterior parietal thalamus ROI and the right inferior temporal cortex, the right precuneus, as well as right cerebellar region 6	No association.
Jiang et al. (2019b)	Seed-based (four hippocampal substructure ROIs = bilateral rostral hippocampus, bilateral caudal hippocampus; Fan et al., 2016), ROI-to-voxels	**MSZ with symptom remission: post-ECT** **>** **pre-ECT:** between the left rostral hippocampus ROI and the left middle temporal gyrus, the left middle frontal gyrus, as well as the left angular gyrus; between the right rostral hippocampus ROI and the left angular gyrus; between the right caudal hippocampus ROI and the left middle temporal gyrus, the right angular gyrus, as well as the right middle frontal gyrus **MSZ no symptom remission: post-ECT** **<** **pre-ECT:** between the left rostral hippocampus ROI and left inferior temporal gyrus; between the left caudal hippocampus ROI and the right inferior temporal gyrus, the left superior temporal gyrus, as well as the post-CG; between the right caudal hippocampus ROI and the right inferior temporal gyrus, the left middle occipital cortex, the left superior temporal gyrus, as well as the right post-CG **MSZ** **>** **DSZ (treatment as usual) baseline:** between bilateral caudal hippocampus ROIs and bilateral superior temporal gyri	**MSZ ECT responders:** The change in connectivity between the left caudal hippocampus and the right angular gyrus was correlated with the general symptoms reduction ratio of the PANSS.
Jiang et al. (2019a)	Seed-based (six insular ROIs = bilateral dorsal anterior insulae, ventral anterior insulae, posterior insulae; Chen et al., 2016), ROI-to-voxels	**MSZ post-ECT** **<** **pre-ECT:** between the left posterior insula ROI and the left middle occipital gyrus; between the right posterior insula and the left orbitofrontal cortex **MSZ** **<** **DSZ (treatment as usual) followup:** between the left posterior insula ROI and the left middle occipital gyrus; between the right posterior insula ROI and the left orbitofrontal cortex **MSZ post-ECT** **>** **HC:** between the right posterior insula ROI and the cerebellum, the thalamus, as well as the post-CG; between the left posterior insula ROI and the thalamus as well as the middle occipital gyrus	**MSZ:** The change of the connectivity between the right posterior insula and the left orbitofrontal cortex was associated with the negative symptoms and general symptoms reduction ratios of the PANSS. The change of the connectivity between the left posterior insula and the left middle occipital gyrus was correlated with the negative symptoms reduction ratio of the PANSS.
Huang et al. (2018)	Graph-based global functional connectivity density of the dorsal medial PFC, ventromedial PFC and left precuneus: number of statistically significant connections between a given voxel and the rest of voxels across the whole brain in a binary network	**MSZ post-ECT** **>** **pre-ECT**: global functional connectivity density of the dorsal medial PFC, the ventromedial PFC, and the left precuneus **MSZ post-ECT** **>** **DSZ (treatment as usual) followup**: global functional connectivity density of the ventromedial PFC	No association
Yang et al. (2020)	Whether pre-ECT connectivity predicts ECT response in regions (23 ROIs = e.g., orbital PFC, superior and mediotemporal lobe, insula, hippocampus, parahippocampal gyrus, amygdala) covered by strong electric fields^*^	**Post-ECT** **<** **Pre-ECT:** between the right amygdala and the left hippocampus	The connectivity change between the amygdala and the hippocampus was positively correlated with the percentage reduction in the PANSS total score. **Prediction:** A regression model constructed using pre-ECT connectivity within regions with strong electric field strength during ECT [i.e., 10 connections within orbital prefrontal lobe, mediotemporal lobe (i.e., hippocampus, parahippocampal gyrus, amygdala) insula, and temporal lobe] generated a good prediction of ECT outcome.

* Supplementary material where all the ROIs should be listed, was not available.

AAL, automated anatomical labeling (Tzourio-Mazoyer et al., 2002); ACC, anterior cingulate cortex; AVH, SCZ individuals with treatment-resistant auditory verbal hallucinations; AVLT, Rey Auditory and Verbal Learning Test; BPRS, Brief Psychiatric Rating Scale; CLZ, clozapine; CPZ, chlorpromazine; DMN, default mode network; DSZ, SCZ individuals taking only antipsychotics; ECT, electroconvulsive therapy; HC, healthy controls; ICA, independent component analysis; MSZ, TRS individuals receiving a regular course of modified ECT combined with antipsychotics; NAVH, non-hallucinating SCZ individuals; NTRS, non-treatment-resistant SCZ; PANSS, Positive and Negative Syndrome Scale; PCC, posterior cingulate cortex, PFC, prefrontal cortex; post-CG, post-central gyrus; preCG, precentral gyrus; ReHo, regional homogeneity; ROI, region-of-interest; SMN, somatomotor network; TRS, treatment-resistant SCZ; UFM, unaffected family members; UTRS, ultra-treatment-resistant SCZ.

The main findings are presented in the following chapters based on the followed treatment regimes. Of note, two studies did not look at or report having studied associations of functional connectivity with clinical scores (Alonso-Solís et al., 2015; McNabb et al., 2018a). Six studies found no association of functional connectivity with clinical variables (Ganella et al., 2017, 2018; Gao et al., 2018; Huang et al., 2018; McNabb et al., 2018b; Wang et al., 2020).

### 3.6. Functional connectivity before the initiation of treatment with riluzole or CLZ

Four studies (three cohorts) could be reviewed for TRS individuals before the initiation of treatment with CLZ or riluzole. In general, they noted frontal hypoconnectivity as follows. Before the start of additional treatment (with riluzole), there was lower functional connectivity in TRS between the bilateral ACC seed ROI and the right anterior PFC compared with healthy controls, and this was associated with lower verbal learning scores in the Rey Auditory and Verbal Learning Test (AVLT; Schmidt, 1996) in TRS (Pillinger et al., 2019). In addition, in the study by McNabb et al. (2018a), CLZ-eligible individuals had higher functional connectivity within the precuneus of the SMN compared to NTRS. Another study noted that corticostriatal connectivities (i.e., between the right dorsal caudate and the right anterior insula as well as the right inferior frontal gyrus) before the initiation of treatment with CLZ could predict CLZ efficacy, i.e., a reduction in positive symptoms post-treatment (Blazer et al., 2022). Furthermore, another study noted that functional connectivity between the basal forebrain and the dorsolateral PFC before the initiation of treatment with CLZ correlated negatively with scores on the Brief Psychiatric Rating Scale (BPRS; Hedlund and Vieweg, 1980; Sarpal et al., 2022a).

### 3.7. Functional connectivity during treatment with riluzole

In one longitudinal study reviewed, an increase in frontal connectivity after riluzole treatment was found. In particular, in individuals with TRS had a higher functional connectivity between the ACC seed ROI and the right anterior PFC post-treatment compared with HC (Pillinger et al., 2019).

### 3.8. Functional connectivity during treatment with CLZ

Based on studies in CLZ-treated patients, it seems that the fronto-temporal hypoconnectivity may be specific for non-responders. Five studies (three separate cohorts) investigated individuals with TRS treated with CLZ and reported consistently lower functional connectivity compared to controls (Ganella et al., 2017, 2018; McNabb et al., 2018b; Blazer et al., 2022; Sarpal et al., 2022a). TRS had lower connectivity predominantly in frontal, temporal, and occipital regions compared to HC (Ganella et al., 2017), and in frontal and temporal regions compared to UFM (Ganella et al., 2018). Lower connectivities were further noted in frontal networks (i.e., cerebello-frontal, cingulo-fronto-temporal, frontoparietal) of UTRS patients in comparison to HC (McNabb et al., 2018b). No study reported higher functional connectivity in TRS currently on CLZ compared to HC. When looking at graph measures, TRS had lower global efficiency and higher local efficiency compared to HC (Ganella et al., 2017). Longitudinal comparisons revealed that the initiation of treatment with CLZ led to an increase in functional connectivities between the right dorsal caudate seed ROI and the right anterior insula, the right inferior frontal lobe, as well as the frontoparietal network in TRS (Blazer et al., 2022). The increase in the mentioned connectivities after 12-weeks of CLZ treatment was associated with a higher percent reduction in positive symptoms (i.e., hallucinations, conceptual disorganization, unusual thought content) of the BPRS. In addition, the connectivity change between the basal forebrain and the dorsolateral PFC correlated positively with the CLZ/N-desmethylclozapine ratio (Sarpal et al., 2022a).

### 3.9. Functional connectivity during mixed treatments

Four studies in SCZ patients who were currently taking either CLZ, other antipsychotics, or a combined treatment with CLZ plus another antipsychotic drug [i.e., (U)TRS] could be reviewed. These studies noted widespread abnormal functional connectivity. It was noted that patients had lower functional connectivities between several brain regions compared to HC. In particular, patients had lower connectivities between thalamic regions, between the DMN and the cingulo-opercular occipital network, between the medial occipital and the mediotemporal networks, between the thalamic region 3 seed ROI and the left intracalcarine cortex, and between the frontoparietal and the mediotemporal as well as the superior frontoparietal networks compared to HC (Kim et al., 2022). This lower connectivity between the frontoparietal and the mediotemporal networks further correlated with the severity of positive, negative, and general symptoms, as well as with the Positive and Negative Syndrome Scale (PANSS; Kay et al., 1987) total score (Kim et al., 2022). Further, patients had lower functional connectivities between the dorsal caudate seed ROI and the sensorimotor cortex, between the ventral-rostral putamen seed ROI and the striatum, and between the ventral striatum seed ROI and the middle frontal gyrus compared to HC (White et al., 2016). In patients, lower functional connectivities between the ventral striatum and the cingulate cortex, the precuneus, as well as the middle frontal gyrus were associated with a higher severity of PANSS positive symptoms (White et al., 2016). The connectivity between the DMN and the mediotemporal network negatively correlated with the severity of negative and general symptoms, and the PANSS total score, and the connectivity between the thalamic region 3 seed ROI and the right lingual gyrus negatively correlated with the severity of all PANSS (sub)scores (Kim et al., 2022). SCZ individuals with treatment-resistant auditory verbal hallucinations (AVH) had lower functional connectivities between the ventromedial PFC seed ROI and bilateral paracingulate cortices, bilateral ACC, as well as bilateral subcallosal cortices, and between the hippocampal formation seed ROI and bilateral PCC as well as bilateral precunei in comparison to HC and non-hallucinating SCZ individuals (NAVH; Alonso-Solís et al., 2015). In comparison to NTRS, (U)TRS had lower functional connectivities between the ventral striatum seed ROI and the substantia nigra, and between the dorsocaudal putamen seed ROI and the pulvinar of the thalamus (White et al., 2016).

Higher functional connectivities in patients compared to HC were noted between the thalamic region 2 seed ROI and the medial occipital network as well as the left precentral gyrus (preCG), between the cingulo-opercular occipital and the medial occipital as well as superior mediotemporal networks, between the mediotemporal and the superior frontoparietal networks, between the thalamic region 1 seed ROI and the left lingual gyrus, between the thalamic region 3 seed ROI and the right supplementary motor cortex, between the thalamic region 6 seed ROI and the frontal medial cortex, the left post-central gyrus (post-CG) as well as the right preCG, and between the thalamic region 9 seed ROI and the left preCG (Kim et al., 2022). In another study, AVH had higher functional connectivities between the posterior inferior parietal lobule seed ROI and bilateral occipital fusiform gyri, bilateral lingual gyri, as well as the left occipital pole, and between the retrosplenial cortex seed ROI and bilateral occipital cortices, bilateral intracalcarine cortices, the left occipital fusiform gyrus, as well as bilateral lingual gyri compared to HC (Alonso-Solís et al., 2015). Higher functional connectivities between the dorsal striatum seeds and the precuneus, the PCC, the medial PFC, the middle temporal gyrus, as well as the inferior and superior parietal lobules were associated with higher PANSS positive subscores (White et al., 2016). In addition, functional connectivity between the thalamic 2 seed ROI and the left preCG positively correlated with the PANSS general symptoms subscore (Kim et al., 2022). In AVH, higher functional connectivities were noted between the dorsomedial PFC seed ROI and bilateral central opercular cortices, bilateral insular cortices, bilateral preCG, as well as bilateral superior temporal gyri, and between the temporal pole seed ROI and the cerebellum compared to HC and NAVH (Alonso-Solís et al., 2015). Furthermore, higher functional connectivities between the dorsal caudate seed ROI and the medial as well as the superior PFC were noted in (U)TRS compared to NTRS (White et al., 2016).

When looking at the local synchronization of rs-fMRI signals, one study (Gao et al., 2018) noted that (U)TRS had higher ReHo in the right middle frontal gyrus (orbital part), the right putamen, bilateral inferior frontal gyri, and bilateral superior medial frontal gyri and lower ReHo in the right fusiform gyrus, bilateral middle occipital gyri / middle temporal gyri, the right superior occipital gyrus, and the right superior parietal lobule compared to HC. The optimal sensitivity and specificity of the ReHo in the left inferior frontal gyrus for differentiating (U)TRS from HC were 100% (17/17) and 86.21% (25/29), respectively. Compared to NTRS, (U)TRS had higher ReHo in the left post-CG, and lower ReHo in the right angular gyrus. Additionally, the ReHo in the left post-CG correctly classified 16 of 17 patients with (U)TRS and 14 of 17 patients with NTRS. The optimal sensitivity for differentiating (U)TRS from NTRS was 94.12%, and the optimal specificity was 82.35%.

It was noted that in (U)TRS, CPZ dosage positively predicted functional connectivities between the dorsal caudate and the lingual gyrus, the cerebellum, the fusiform gyrus, as well as the occipital lobe, between the dorsocaudal putamen and the lingual gyrus as well as the cuneus, and between the ventral-rostral putamen and the medial frontal gyrus. CPZ dosage also negatively predicted functional connectivities between the ventral striatum and the PCC, the lingual gyrus, the middle frontal gyrus, as well as the cerebellum, and between the dorsal caudate and the post-CG in (U)TRS (White et al., 2016).

### 3.10. Functional connectivity in TRS before the initiation of treatment with ECT

Widespread abnormal functional connectivity was present before the initiation of treatment with ECT. Seven studies (with two patient cohorts) were reviewed to cover this topic. In ECT-eligible TRS individuals, higher functional connectivities were noted between the left angular and the right middle temporal gyri (Hu Q. et al., 2022), and between the left superior occipital cortex and the left rostral temporal thalamus seed ROI as well as the right caudal temporal thalamus seed ROI (Wang et al., 2020) compared to HC. The same cohort of patients exhibited higher functional connectivities between bilateral caudal hippocampus seed ROIs and bilateral superior temporal gyri compared to DSZ (Jiang et al., 2019b). One study did not find any significant differences between patients and control cohorts before the initiation of treatment with ECT (Huang et al., 2018), and two studies did not differentiate between MSZ and DSZ but investigated them as a single group (Jiang et al., 2019a; Hu H. et al., 2022). These studies did not report any associations to pre-treatment clinical scores.

Although the second ECT cohort (Yang et al., 2020) had no control cohorts to compare to, the authors investigated predictive values of functional connectivity within the patient sample. They noted that a regression model constructed using functional connectivity before the initiation of treatment with ECT within regions with strong electric field strength during ECT generated a good prediction of outcome. Specifically, the predictive connectivities were noted between the left inferior orbitofrontal cortex and the left insula as well as the left inferior temporal lobe, between the right insula and the left middle orbitofrontal cortex as well as the left inferior temporal lobe, between the right amygdala and the left hippocampus as well as the right parahippocampal gyrus, between the right superior temporal pole and the left insula as well as the middle temporal lobe, between the left middle temporal pole and the left parahippocampal gyrus, and between the right middle temporal pole and the left hippocampus.

### 3.11. Functional connectivity in TRS after treatment with ECT

In general, the reviewed studies noted ECT-induced effects specifically on the limbic connectivity. A decrease in functional connectivity was noted post-ECT compared to pre-treatment between the right amygdala and the left hippocampus, and this correlated positively with the percentage reduction in the PANSS total score (Yang et al., 2020). Post-ECT, reduced connectivities were found between the left rostral temporal thalamus seed ROI and the left superior occipital cortex, between the left caudal temporal thalamus seed ROI and the left middle frontal cortex, and between the right caudal temporal thalamus seed ROI and the left superior occipital cortex compared to pre-treatment (Wang et al., 2020).

Increased functional connectivities post-ECT compared to pre-treatment were noted between the right sensory thalamus seed ROI and the right putamen (Wang et al., 2020), between the right angular and the left middle temporal gyri (Hu Q. et al., 2022), and between several DMN and cerebellar regions (Hu H. et al., 2022).

Several associations to clinical scores were found. The higher functional connectivity between the left angular and the right middle temporal gyri correlated with the reduction of the PANSS general symptoms subscore (Hu Q. et al., 2022). In addition, the connectivity change (pre- vs. post-treatment) between the right angular and the right middle temporal gyri correlated with reductions of the PANSS negative and total (sub)scores (Hu Q. et al., 2022), and the connectivity change between the right posterior insula and the left orbitofrontal cortex positively correlated with a reduction in general and negative symptoms of the PANSS, and the connectivity change between the left posterior insula and the left middle occipital gyrus positively correlated with an improvement of PANSS negative symptoms (Jiang et al., 2019a). Further, the connectivity change between the left middle temporal gyrus and the left cerebellar crus region 2 positively correlated with improvements in the PANSS general symptoms and total (sub)scores (Hu H. et al., 2022). The connectivity changes between the left inferior temporal gyrus and the left cerebellar crus region 2, as well as between the right angular gyrus and the left cerebellar region 45 positively correlated with an improvement of PANSS general symptoms (Hu H. et al., 2022).

In refractory MSZ, functional connectivities increased post-ECT between the right posterior parietal thalamus seed ROI and the right inferior temporal cortex as well as the right cerebellar region 6 compared to pre-treatment (Wang et al., 2020). In MSZ with no symptom remission, functional connectivities decreased post-ECT between the left rostral hippocampus seed ROI and the left inferior temporal gyrus, between the left caudal hippocampus seed ROI and the right inferior temporal gyrus, the left superior temporal gyrus, as well as the post-CG, and between the right caudal hippocampus seed ROI and the right inferior temporal gyrus, the left middle occipital cortex, the left superior temporal gyrus, as well as the right post-CG compared to pre-treatment (Jiang et al., 2019b). In non-refractory MSZ, functional connectivities decreased post-ECT between the right posterior parietal thalamus seed ROI and the right inferior temporal cortex, the right precuneus, as well as the right cerebellar region 6 compared to pre-treatment (Wang et al., 2020). In MSZ with symptom remission, functional connectivities increased post-ECT between the left rostral hippocampus seed ROI and the left middle temporal gyrus, the left middle frontal gyrus, as well as the left angular gyrus, between the right rostral hippocampus seed ROI and the left angular gyrus, and between the right caudal hippocampus seed ROI and the left middle temporal gyrus, the right angular gyrus, as well as the right middle frontal gyrus compared to pre-treatment (Jiang et al., 2019b). In individuals who responded to modified ECT, the connectivity change between the left caudal hippocampus and the right angular gyrus was associated with the general symptomatology reduction ratio of the PANSS (Jiang et al., 2019b).

Graph theoretical approaches noted nodal changes in 22 brain regions post-ECT compared to pre-treatment, which were mainly located in the frontal lobe and the cerebellum (Hu H. et al., 2022). Post-ECT, increased global functional connectivity densities of the dorsal medial PFC, the ventromedial PFC, and the left precuneus were noted compared to pre-treatment (Huang et al., 2018). In addition, local efficiency increased, and global efficiency decreased compared to pre-treatment (Hu H. et al., 2022).

The studies found differences in (U)TRS in comparison with control cohorts. Post-ECT, higher functional connectivities were noted in MSZ between the right posterior insula seed ROI and the cerebellum, the thalamus, as well as the post-CG, between the left posterior insula seed ROI and the thalamus as well as the middle occipital gyrus (Jiang et al., 2019a), between ROIs of the right angular and the left middle temporal gyri, and between ROIs of the left angular and the right middle temporal gyri (Hu Q. et al., 2022) compared with HC. In comparison with DSZ, MSZ had higher functional connectivity between the left angular and the right middle temporal gyri, and between the right angular and the left middle temporal gyri (Hu Q. et al., 2022) and lower connectivity between the left posterior insula seed ROI and the left middle occipital gyrus, as well as between the right posterior insula seed ROI and the left orbitofrontal cortex (Jiang et al., 2019a).

ECT treated patients had higher global functional connectivity density of the ventromedial PFC compared with DSZ (Huang et al., 2018).

### 3.12. Multimodal MRI

Some of the studies reviewed combined rs-fMRI results with additional imaging findings, i.e., with striatal tissue iron measures as studied with R2^*^ mapping during CLZ treatment (Blazer et al., 2022), with neurometabolite levels of the ACC as studied with ^1^H-MR spectroscopy as well as regional cerebral blood flow information from arterial spin labeling sequences during riluzole treatment (Pillinger et al., 2019), and hippocampal (Jiang et al., 2019b) as well as insular (Jiang et al., 2019a) volumetric information from structural MR sequences during ECT.

A CLZ study did not observe any significant relationship between changes in striatal tissue iron measures and CLZ efficacy, neither were there any correlations to the connectivity findings (Blazer et al., 2022).

The study that combined neurometabolite levels and regional cerebral blood flow information (Pillinger et al., 2019) with connectivity findings noted that pre-riluzole treatment glutamate plus glutamine (Glx) levels correlated positively with the negative symptom severity of the PANSS and negatively with verbal learning scores assessed by the AVLT in TRS. Riluzole decreased Glx levels and increased the connectivity between the ACC and the anterior PFC in TRS relative to HC. Riluzole did not alter regional cerebral blood flow.

An ECT study looked into hippocampal volumetry in addition to functional connectivity (Jiang et al., 2019b). Baseline results showed that non-responders had lower volumes in the left hippocampus-amygdala transition area compared with responders and HC, that both non-responders and responders had lower volumes in the right hippocampus-amygdala transition area compared with HC, and that responders had higher volumes in the cornu ammonis area 4 bilaterally compared with HC. ECT induced significant volume increases in bilateral hippocampi and hippocampal subfields in both responding and non-responding MSZ. In responders, a significant association was observed between the left cornu ammonis area 4 volume increase and the general symptoms reduction ratio of the PANSS.

Another ECT study looked for associations between the changes of insular volumetry and functional connectivity (Jiang et al., 2019a). In MSZ, they found increased volumes in bilateral posterior insulae post-ECT. The volume increase in the right posterior insula was associated with reductions of PANSS positive and general symptoms as well as the PANSS total score in MSZ. In addition, increased volume in the left posterior insula was associated with reduced functional connectivity between the left posterior insula and the right middle temporal gyrus.

## 4. Discussion

In this review, we investigated rs-fMRI findings and their associations to clinical symptoms in TRS and UTRS from the last decade in order to look for support to the hypothesis that SCZ is a brain dysconnection syndrome (Stephan et al., 2009). The 18 studies reviewed included treatment with CLZ (and/or mixed medication), riluzole, and ECT. They showed widespread functional connectivity abnormalities in (U)TRS and some of this variability was linked to symptoms, treatment response, and multimodal image findings. Predictions based on functional connectivity, CPZ-equivalent dosage, and symptom scores were also presented.

### 4.1. The relevance of rs-fMRI sequence designs and connectivity methods

Most of the studies had cross-sectional designs where TRS patients had already been exposed to antipsychotic medication for a longer while (Alonso-Solís et al., 2015; White et al., 2016; Ganella et al., 2017, 2018; Gao et al., 2018; McNabb et al., 2018b; Kim et al., 2022). Therefore, it was not possible to investigate possible functional connectivity changes induced by CLZ treatment based on the aforementioned studies. Treatment response to CLZ was studied in one longitudinal TRS cohort, however, the design of that study did not include any control cohorts (Blazer et al., 2022; Sarpal et al., 2022a).

The rs-fMRI sequence designs (e.g., length, instructions given) and different preprocessing methods applied (e.g., fieldmap correction) could have affected the functional connectivity results obtained. Some studies applied more advantageous multiband fMRI sequences (McNabb et al., 2018a; Blazer et al., 2022; Sarpal et al., 2022a) and an additional fieldmap sequence for distortion corrections (McNabb et al., 2018a). In addition, some studies benefited from acquiring more information by the application of longer duration multiband (Blazer et al., 2022; Sarpal et al., 2022a) and multiecho (Pillinger et al., 2019) fMRI sequences where more acquisitions can be made in the same timeframe in comparison with traditional fMRI sequences and where the reliability of connectivity measures is improved (Lynch et al., 2020).

Most of the studies reviewed applied seed-based methods (n=10) where a priori hypothesis on regions with connectivity abnormalities are given. Although the exact selection of the seed locations may have affected the results, all the studies reviewed based their seed selections on previous research or predefined atlases and graph-based analyses applied the widely used AAL atlas (Tzourio-Mazoyer et al., 2002). Thus, analyses are repeatable and findings can be compared to studies that applied the same atlases or regions.

### 4.2. Frontal hypoconnectivity before the initiation of treatment with CLZ or riluzole

According to the dysconnectivity hypothesis (Stephan et al., 2009) functional communication between regions of the brain is affected in SCZ. At a later stage of the disorder, this may lead to structural abnormalities. We reviewed TRS studies before the initiation of treatment with CLZ or riluzole in individuals who had not responded to at least two antipsychotic trials. TRS had higher connectivity in the precuneus as part of the SMN compared to NTRS (McNabb et al., 2018a) and lower connectivities within frontal regions (i.e., ACC, PFC) compared to HC (Pillinger et al., 2019). In particular, it was noted that connectivities within frontal brain regions (i.e., ACC, PFC, inferior frontal gyrus, basal forebrain) correlated with verbal learning scores assessed by the AVLT (Pillinger et al., 2019) and psychosis symptoms assessed by the BPRS (Sarpal et al., 2022a). It was also observed that corticostriatal connectivities (i.e., between the right dorsal caudate and the right anterior insula as well as the right inferior frontal gyrus) before the initiation of treatment may predict CLZ efficacy in TRS, i.e., a reduction in positive symptoms (Blazer et al., 2022). Such abnormalities in the frontal brain regions, especially in the PFC, are consistent with previous SCZ literature (Zhou et al., 2015) and the dysconnectivity hypothesis. Interestingly, previous dynamic causal modeling studies reported abnormal directed connectivity involving the PFC in SCZ (Friston et al., 2016). This supports the idea that functional connectivity originating from the PFC causes abnormal communication. Although it is not possible to rule out the effects of antipsychotics based on the studies reviewed, previous studies have found connectivity irregularities in the PFC in drug-naïve first-episode SCZ as well as in individuals with a history of short duration of antipsychotic medication use in first-episode SCZ (Mwansisya et al., 2017). Although these findings support frontal hypoconnectivity in TRS, the conclusions that can be made are limited, as only one of the studies (Pillinger et al., 2019) made comparisons of TRS (*n* = 19) with HC (*n* = 17) and only one of them (McNabb et al., 2018a) made comparisons of TRS (*n* = 15) with NTRS (*n* = 10) with limited sample sizes.

### 4.3. Increase in frontal connectivity during treatment with riluzole

The within frontal brain regions connectivity (i.e., ACC-PFC) was noted to increase above the levels of HC after treatment with riluzole (Pillinger et al., 2019). As this study focused solely on functional connectivity differences of the ACC, further conclusions in terms of PFC or other brain regions cannot be drawn. Unfortunately, post-riluzole clinical scores were not collected, neither did the authors include NTRS for comparison. Accordingly, the follow-up findings cannot be specifically attributed to TRS.

### 4.4. Fronto-temporal connectivity during treatment with CLZ

Five rs-fMRI studies with CLZ monotherapy treatment were identified (Ganella et al., 2017, 2018; McNabb et al., 2018b; Blazer et al., 2022; Sarpal et al., 2022a). Among them, two TRS cohorts (Ganella et al., 2017, 2018; McNabb et al., 2018b) were compared to control cohorts. Both of these studies used the AAL atlas to study brain connectivity and should therefore be comparable. However, different treatment responses may explain some of the inconsistencies found. McNabb et al. (2018b) specified a UTRS subgroup and noted that they presented with lower connectivity between cerebellar-frontal, cingulo-frontal-temporal, and frontoparietal regions compared to HC. As no significant differences were found in TRS compared to UTRS and HC (McNabb et al., 2018b), this supports the idea that in treatment-responsive TRS the connectivity may have returned to a normalized level somewhere between the connectivity levels of UTRS and HC after the initiation of treatment with CLZ. However, the other research group reported lower functional connectivity between fronto-temporal, fronto-occipital, temporo-occipital, and temporo-temporal brain regions in TRS compared to HC (Ganella et al., 2017) and in frontal and temporal regions compared to UFM (Ganella et al., 2018). It is important to note that they, however, did not specify whether the TRS cohort had responded to CLZ treatment, although this is rather likely in view of the relatively low mean PANSS total score. Rather differing CPZ-equivalent doses (615.4 ± 55.84; Ganella et al., 2017, 2018) compared to the other study (McNabb et al., 2018b) with lower CPZ equivalents in TRS (359.2 ± 275.5) and higher CPZ-equivalents in UTRS (772.1 ± 522.6) were reported though. Therefore, some of the mentioned connectivity differences noted in fronto-temporal regions in TRS during CLZ treatment could be partly driven by UTRS patients in the study by Ganella et al. (2017, 2018). This, however, does not rule out the possibility that connectivity abnormalities may remain in treatment responders, although in somewhat normalized levels. Indeed, no associations of functional connectivity to clinical symptoms were noted in the studies reviewed. Whether hypoconnectivity is specific for non-responding individuals remains unanswered. However, in line with the dysconnectivity hypothesis, there is previous evidence for functional abnormalities between frontal and temporal regions in SCZ (Friston et al., 2016).

A higher local efficiency in TRS compared to HC was noted (Ganella et al., 2017) which suggests a topological organization indicative of segregated neural processing (Rubinov and Sporns, 2010). This is in line with other studies in SCZ (Hadley et al., 2016) suggesting that functional integration and segregation can be modulated with antipsychotic medications, but only in those who respond to treatment.

Only two of the studies reviewed were longitudinal and consisted of a TRS cohort overlap (Blazer et al., 2022; Sarpal et al., 2022a). Interestingly, in these seed-based studies (i.e., seeds in the striatum, the basal forebrain, the PFC, and bilateral hippocampi) connectivity increases between the striatum (i.e., dorsal caudate) and the frontal lobe, the insula, as well as the frontoparietal network correlated with a reduction of psychotic symptoms (Blazer et al., 2022). Moreover, connectivity changes within frontal regions (between the basal forebrain and the dorsolateral PFC) correlated with the CLZ/N-desmethylclozapine ratio (Sarpal et al., 2022a). Specifically, response to CLZ as well as higher CLZ/N-desmethylclozapine ratios were associated with increases in functional connectivity (Blazer et al., 2022; Sarpal et al., 2022a). Therefore, the suggested fronto-temporal hypoconnectivity during CLZ treatment may be specifically related to non-response.

### 4.5. Widespread abnormal functional connectivity during mixed treatments

Probably due to the high heterogeneity in the patient cohorts concerning medications and other clinical variables (e.g., lifetime antipsychotic drug exposure, duration of illness, treatment adherence), widespread abnormalities in connectivity were noted. Three of the mixed-treatment studies (Alonso-Solís et al., 2015; White et al., 2016; Kim et al., 2022) applied seed-based methods (i.e., thalamus, striatum, PCC, PFC, temporo-parietal junction, temporal cortex, hippocampi, parahippocampi, retrospenial cortex). Among these studies, several regions (i.e., superior frontoparietal network, striatum, PFC, PCC, precuneus, mediotemporal network, thalamus, cingulo-opercular occipital network, medial occipital network, intracalcarine cortex) showed both higher and lower connectivities in (U)TRS on mixed treatments compared to HC and NTRS (Alonso-Solís et al., 2015; White et al., 2016; Kim et al., 2022). The noted connectivity between the frontoparietal and the mediotemporal networks correlated negatively with all PANSS (sub)scores (Kim et al., 2022). This further supports the dysconnectivity hypothesis with fronto-temporal abnormalities (Friston et al., 2016) which could be related to differences in individual treatment response.

It was also observed that CPZ-equivalents could negatively predict functional connectivities between the ventral striatum and DMN regions (i.e., PCC, middle frontal gyrus), the lingual gyrus, as well as the cerebellum, and between the dorsal caudate and the post-CG in (U)TRS (White et al., 2016). It is interesting to note that further negative correlations were shown in the connectivities between the ventral striatum seed and DMN regions and positive symptoms (White et al., 2016). Similarly, CPZ-equivalents in (U)TRS positively predicted connectivities between the dorsal caudate and the lingual gyrus, the cerebellum, the fusiform gyrus, as well as the occipital lobe, between the dorsocaudal putamen and the lingual gyrus as well as the cuneus (part of the DMN), and between the ventral-rostral putamen and the medial frontal gyrus (part of the DMN). Further, higher connectivities between the dorsal striatum seeds and DMN regions, the middle temporal gyrus, as well as the inferior and superior parietal lobules were associated with higher PANSS positive subscores (White et al., 2016). Another study found negative correlations between the DMN and the mediotemporal networks connectivity and negative and general symptoms, as well as the PANSS total score (Kim et al., 2022). Interestingly, in the reviewed studies, CPZ equivalents could be used to predict connectivity between the striatum and the DMN which further correlated with symptoms along with associations with DMN-temporal network connectivity. As these participants were on mixed medications, some of the connectivity abnormalities may be caused by antipsychotic medication affecting the dopamine system of the striatum (McCutcheon et al., 2019), which may in turn modulate DMN connectivity (Hu et al., 2017). The effect may also partly be explained by glutamatergic signaling in CLZ-responders, although the cohorts likely consisted of mixed responders.

The connectivity between the thalamus and the lingual gyrus correlated negatively with all PANSS (sub)scores (Kim et al., 2022). Positive correlation was also found between the thalamus—preCG connectivity and the PANSS general symptoms subscore (Kim et al., 2022). In line with that study, abnormalities of thalamo-cortical connectivity were noted previously in SCZ, including higher connectivity between the thalamus and the sensorimotor cortex (Pergola et al., 2015). In fact, the thalamus has been suggested to be involved in the pathogenic mechanisms underlying the psychotic symptoms of SCZ (Jiang et al., 2021). However, there has been inconsistencies in the findings, and it was suggested that thalamic subregions should be specifically studied (Pergola et al., 2015). Kim et al. (2022) applied nine thalamic seeds and found connectivity differences between thalamic and frontal brain regions between TRS and HC, which, however, did not correlate with symptoms. As the TRS cohort may have consisted of both treatment responders and non-responders, their brain connectivity might rely on different mechanisms and separate investigations of these subgroups may be necessary.

Although Alonso-Solís et al. (2015) did not find any significant correlations between connectivity and clinical scores, they defined AVH and NAVH subgroups and reported significantly lower connectivities in AVH between the PFC and the cingulate as well as the subcallosal cortices, and between the hippocampus and the bilateral PCC as well as the precunei. In addition, they noted higher connectivity between the PFC and the central opercular cortices, the insular cortices, bilateral preCG, as well as the temporal gyri, and between the temporal pole and the cerebellum in AVH compared to NAVH. In fact, there is some previous evidence that prefrontal-temporal connectivity and specifically hippocampal regions may contribute to hallucinations (Lawrie et al., 2002; Cachia et al., 2020).

When looking at local connectivity, (U)TRS had lower ReHo in the right fusiform gyrus, bilateral middle occipital gyri / middle temporal gyri, the right superior occipital gyrus, and the right superior parietal lobule, and higher ReHo in the right middle frontal gyrus, the right putamen, bilateral inferior frontal gyri, and bilateral superior medial frontal gyri compared to HC (Gao et al., 2018). In fact, the ReHo in the left inferior frontal gyrus could reliably differentiate (U)TRS from HC (Gao et al., 2018). In addition, (U)TRS had lower ReHO in the right angular gyrus compared to NTRS (Gao et al., 2018), and higher ReHo in the left post-CG which could differentiate nearly all (U)TRS patients from NTRS patients (Gao et al., 2018).

In conclusion, widespread connectivity abnormalities were found specifically in frontal, thalamic, and hippocampal regions, as well as in regions of the DMN and SMN, and between frontal and temporal regions in TRS.

### 4.6. Functional connectivity in TRS before the initiation of treatment with ECT

Only two different patient cohorts investigating functional connectivity are reported on the included ECT studies. Study participants on these studies were on mixed treatments, which partly included CLZ. Therefore, some of them might have been CLZ-resistant while others were not. These mixed treatment and response subgroups may explain some of the widespread functional connectivity irregularities pre-ECT. The study which included comparisons to control cohorts showed higher connectivity in (U)TRS (Jiang et al., 2019b; Wang et al., 2020; Hu Q. et al., 2022). In particular, (U)TRS had higher connectivities between the angular gyrus and temporal regions, between thalamic and occipital regions, and between hippocampal and other temporal regions compared to HC (Wang et al., 2020; Hu Q. et al., 2022). Although no differences in frontal connectivity were reported, the other regions with noted irregularities (e.g., temporal, occipital, thalamus) are in line with our previous discussion. In addition, higher connectivity was shown between temporal and mediotemporal (i.e., hippocampal) regions in MSZ compared to DSZ (Jiang et al., 2019b). However, here the DSZ cohort consisted of individuals who were not necessarily first-line responders, e.g., they were on antipsychotics instead of ECT but the reason for this could also have been refusal of ECT. Therefore, a clear distinction to this control cohort with regard to treatment non-response is not possible. Unfortunately, the other study (Yang et al., 2020) did not include any control cohorts for comparison. However, their regression model using functional connectivity before the initiation of treatment within regions with strong electric field strength during ECT found ten connections (i.e., fronto-insular, fronto-temporal, insular-temporal, temporal-temporal) which generated a good prediction of outcome (Yang et al., 2020). This is in line with previous reports on fronto-temporal connectivity predictions in individuals with major depression treated with ECT (Leaver et al., 2018).

### 4.7. Functional connectivity changes induced by ECT

ECT has the possibility to modulate brain connectivity, which may lead to symptom improvement. We reviewed two patient cohort studies that investigated rs-fMRI post-ECT. However, they were not completely comparable due to different approaches. Yang et al. (2020) specifically studied regions which were covered by strong electric fields during bitemporal ECT and reported on decreased connectivity between regions of the mediotemporal lobe (i.e., right amygdala and hippocampus) post-ECT compared to pre-treatment and this correlated with a reduction in the PANSS total score (Yang et al., 2020). Functional changes in these structures following ECT have been reported in previous SCZ studies (Thomann et al., 2017). Further, there is robust evidence on structural changes of the limbic system (e.g., hippocampi, amygdala) induced by ECT in major depression as well as in SCZ (Moon et al., 2021; Leaver et al., 2022). In fact, it has been suggested that ECT improves symptoms by correcting hippocampal-amygdala dysfunction through the action of cerebello-thalamo-cortical circuits (Leaver et al., 2022). Modulation of hippocampal connectivity in association with symptom reduction induced by ECT would be consistent with both dopamine and glutamate hypotheses of SCZ (Moon et al., 2021). However, the relevance of (well-replicated) hippocampal changes induced by ECT is not completely understood (Leaver et al., 2022).

The second cohort consisted of separate articles applying multiple analysis methods and seed ROIs (Huang et al., 2018; Jiang et al., 2019a,b; Wang et al., 2020; Hu H. et al., 2022; Hu Q. et al., 2022) which together reported both decreased and increased connectivities post-ECT, changes in graph measures, as well as connectivity differences between groups which varied depending on response to bitemporal ECT. When looking at the correlations to symptoms, it was noted that increased connectivity between the left angular and the right middle temporal gyri, and decreased connectivity between the right angular gyrus and the cerebellar region 45 were correlated with reductions of PANSS general and negative symptoms, and of the PANSS total score (Hu H. et al., 2022; Hu Q. et al., 2022). Moreover, the decreased connectivities between bilateral posterior insulae and the left orbitofrontal cortex as well as the left middle occipital gyrus correlated with an improvement of negative symptoms (Jiang et al., 2019a). The decreased connectivities between the right posterior insula and the left orbitofrontal cortex (Jiang et al., 2019a), and between the cerebellar crus region 2 and the left middle temporal as well as the left inferior temporal gyri correlated with reductions in PANSS general symptoms (Hu H. et al., 2022). Further, the decreased connectivity between the left middle temporal gyrus and the left cerebellar crus region 2 was associated with reduction in PANSS total score (Hu H. et al., 2022). Although these studies found no correlations to positive symptom reductions, it seems that fronto-temporo-occipital regions, the insula, and the cerebellum are involved in the symptomatology of (U)TRS. We have previously discussed the relevance of frontal and temporal regions in SCZ. In addition, a recent study noted abnormalities in cerebellar connectivity in first-episode SCZ in correlation to symptoms (Feng et al., 2022). Furthermore, structural changes of the insula, a part of the limbic system, were previously noted by both of these research groups (Wang et al., 2019; Gong et al., 2020) who now presented functional changes induced by ECT. Such findings support previous results on both structural and functional changes of the insula following ECT (Thomann et al., 2017; Leaver et al., 2022). ECT may induce functional (and structural) changes which further lead to symptom reduction in SCZ.

In line with this, it has been suggested that networks relevant to seizure physiology (involving, for example, the thalamus and the cerebellum) are important for ECT outcomes (Leaver et al., 2022). In fact, decreased connectivities between the thalamus and inferior temporal regions, the precuneus, as well as the cerebellum were noted in non-refractory MSZ (Wang et al., 2020). Moreover, in line with well-known hippocampal changes following ECT, increased connectivities between the hippocampus and the middle temporal, the middle frontal, as well as the angular gyri were noted in MSZ with symptom remission (Jiang et al., 2019b). In addition, the connectivity change between the left caudal hippocampus and the right angular gyrus was associated with PANSS general symptom reduction (Jiang et al., 2019b).

There were nodal changes (i.e., efficiency, betweenness centrality, degree) in the frontal lobe and the cerebellum post-ECT (Hu H. et al., 2022), although the authors did not state the directions of the changes. Post-ECT, global functional connectivity densities of the dorsal medial PFC, the ventromedial PFC, and the left precuneus increased (Huang et al., 2018) but no associations to clinical scores were found. In addition, local efficiency increased, and global efficiency decreased post-ECT (Hu H. et al., 2022). It seems, however, that associations of these local and global measures to clinical symptoms were not studied. Although it was noted that the global functional connectivity density of the ventromedial PFC was higher in MSZ patients post-ECT compared to DSZ (Huang et al., 2018), these findings may solely reflect differences between ECT and antipsychotic treatment and not response-related characteristics per se.

### 4.8. Multimodal MRI findings

Functional regions showing abnormal connectivity according to the reviewed studies are in line with findings from multimodal MR studies in TRS. However, it is good to keep in mind that although anatomical abnormalities are common in SCZ, they seem to evolve over time as a consequence of the disorder (Friston et al., 2016). Therefore, it is likely to see widespread changes in TRS patients with a relatively long duration of illness. Interestingly, however, the reviewed studies that combined multimodal MRI found associations between metabolic and structural brain abnormalities and changes in functional connectivity.

A study that combined information on neurometabolite levels and functional connectivity noted that riluzole (a glutamate modulator) decreased Glx levels in the ACC and increased functional connectivity between the ACC and the anterior PFC in TRS relative to HC (Pillinger et al., 2019). In fact, previous studies have implicated metabolic changes in frontal brain regions and the striatum (as studied by MR spectroscopy) which were associated with treatment response and clinical symptoms (Goldstein et al., 2015; Mouchlianitis et al., 2016; Iwata et al., 2019; Tarumi et al., 2020; McQueen et al., 2021).

In the other study reviewed (Jiang et al., 2019b), ECT responders had lower volumes in the right hippocampus-amygdala transition area and higher volumes in bilateral hippocampal areas (i.e., cornu ammonis area 4) at baseline compared to HC. This was in line with the finding on ECT responders who exhibited increased connectivities between the hippocampus and the PFC as well as regions of the DMN post-treatment. In the same cohort, an increased volume of the left posterior insula correlated negatively with functional connectivity between the left posterior insula and the right middle temporal gyrus (Jiang et al., 2019a). In fact, other structural MRI studies revealed widespread reductions in thickness in several cortical (e.g., frontal, parietal, temporal, occipital) and subcortical structures (e.g., striatum [nucleus accumbens, putamen], thalamus, mediotemporal lobe [amygdala, hippocampus]) in TRS compared to HC and NTRS (Zugman et al., 2013; Liu et al., 2022), and in UTRS compared to HC and first-line responders (Shah et al., 2020). Similar regions have now been associated with functional changes in the reviewed studies. In particular, although treatment with CLZ was associated with global cortical thinning and reductions in the medial PFC and the periventricular area volumes regardless of treatment response (Ahmed et al., 2015), other studies noted that larger temporal and prefrontal volumes and thinner frontal cortices before the initiation of treatment correlated with the improvement of symptoms post-treatment (Arango et al., 2003; Molina et al., 2003, 2014). Treatment with CLZ was associated with a reduction in the caudate volume in correlation with clinical improvement (Scheepers et al., 2001; Tronchin et al., 2020) and further correlated with caudal N-acetylaspartate level reductions as studied by MR spectroscopy (Krajner et al., 2022). In addition, reductions of the thalamus and putamen volumes were associated with symptom improvement (Tronchin et al., 2020). Treatment-related reductions were further noted in the hippocampal volumes, as well as in the thickness of the temporal and caudal middle frontal cortices (Tronchin et al., 2020; Krajner et al., 2022).

The reviewed studies did not combine any measures from diffusion tensor imaging which can be used to assess the directional preference of diffusion (i.e., fractional anisotropy [FA]), diffusion rates (e.g., radial diffusivity), and white matter (WM) connectivity in the brain. However, long-term functional abnormalities may lead to changes in the WM tracts. Moreover, previous SCZ studies suggested that lower FA and greater radial diffusivity values in the corpus callosum exist as biomarkers for TRS with association to the severity of symptoms (McNabb et al., 2018c). In fact, FA values in several brain tracts were lower in individuals with TRS compared to HC and increased after 12 weeks of treatment with CLZ compared to pre-treatment (Ozcelik-Eroglu et al., 2014). In line with this, after 6 months of switching to CLZ, individuals with TRS displayed a reduction of FA values in the corpus callosum and in the anterior and superior corona radiata compared to HC (Tronchin et al., 2021). Similarly, another study reported lower FA values in multiple tracts (e.g., corona radiata, corpus callosum, superior longitudinal and uncinate fasciculi) with correlations to symptoms in TRS compared to HC and NTRS (Ochi et al., 2020). However, another study reported higher FA and radial diffusivity values in individuals treated with CLZ in the prefronto-subcortical and fronto-subcortical tracts compared to SCZ individuals on first-generation antipsychotics (Leroux et al., 2018). In CLZ-treated and never-treated individuals, a relatively stronger disrupted FA organization of WM structural networks as well as decreased graph connectivity measures (e.g., in the thalamus, the hippocampus, prefrontally, occipitally) were found compared to risperidone-treated individuals with associations to cognitive function (Luo et al., 2020). Individuals diagnosed with TRS showed a higher WM regional vulnerability index compared to individuals with treatment-responsive SYZ (Kochunov et al., 2019). In summary, in addition to the functional abnormalities reported in the reviewed studies, treatment-related effects on WM connectivity in SCZ have been suggested in other studies that applied diffusion imaging.

## 5. Limitations

This review summarized rs-fMRI findings from the last decade to provide an overview of the current knowledge on brain functional connectivity abnormalities and their associations to symptoms in TRS and UTRS and looked for support for the dysconnection hypothesis in SCZ. However, our methods and the ones from the reviewed articles were not without limitations. First, we limited the time window search to the last decade, and therefore any previous findings that may have supported or provided additional information were ignored. However, we have looked for support for the presented findings from additional literature. Furthermore, although we included studies which specifically defined TRS as having failed at least two adequate treatment episodes with different antipsychotic drugs, we might have excluded studies which in fact included TRS patients but did not provide this information in the methods section. In addition, we did not carry out meta-analysis due to the heterogeneity in the methods applied in the reviewed studies. In fact, the different analytical approaches applied in the reviewed studies may not be comparable and may measure different phenomena, which limits the possibility for meta-analysis. In addition, we have not defined symptom severity or antipsychotic dosage limits due to inconsistencies in the reviewed studies.

As many of the included studies were cross-sectional and did not include any “baseline” acquisitions in TRS before the initiation of treatment with CLZ, the differences noted could partly be related to different drug treatments in TRS and NTRS patients and not to differences in the subgroups of the disorder *per se*. Furthermore, trial durations, CPZ equivalent doses, lifetime antipsychotic drug exposure, duration of illness, and treatment adherence limit the generalizability of the findings. For example, response to CLZ has been shown to depend on the time of initiation (Yoshimura et al., 2017). This could eventually lead to more persistent irregularities in brain function as well as structure. In addition, functional irregularities could differ between individuals with initial or later onset TRS.

The major limitation, though, was that most of the studies did not differentiate between TRS and UTRS and investigated heterogeneous patients cohorts treated with mixed treatments (with or without CLZ). This is critical as in different subtypes of the disorder an interplay between dopaminergic and glutamatergic pathways involving frontal, striatal, and temporal brain regions in separate ways is likely. Although many of the studies defined both TRS and NTRS for group comparisons, it was often unclear whether study participants were responding to their current treatment (e.g., CLZ or ECT combined with antipsychotics). Future studies should, for example, define response as a certain percentage PANSS reduction. One cross-sectional study on CLZ treatment defined UTRS in addition to TRS (McNabb et al., 2018b), and two ECT studies reported results on ECT responders and non-responders (Jiang et al., 2019b; Wang et al., 2020). Others reported results based on covariation of treatment response, probably due to low sample sizes, which would have limited the power to study UTRS separately. It is likely that the ECT cohorts included individuals who were not responding to previous CLZ treatment, i.e., UTRS, and participants who never had a trial of CLZ, which further limits the generalizability of the findings.

In addition, differences in sequence designs (e.g., length, instructions given) could have led to variations in functional connectivity results that cannot be controlled for.

## 6. Future directions

**Figure 3:**
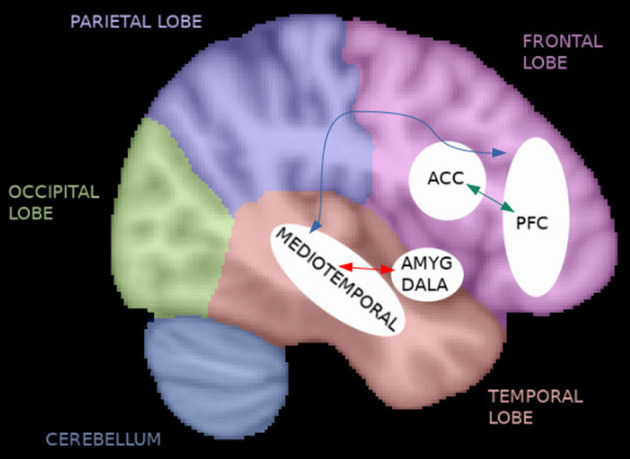
Functional connectivity in TRS. The reviewed studies noted frontal hypoconnectivity before the initiation of treatment with clozapine (CLZ) or riluzole (e.g., anterior cingulate cortex [ACC] and prefrontal cortex [PFC]), an increase in frontal connectivity after riluzole treatment (green arrow), fronto-temporal hypoconnectivity that may be specific for non-responders (blue arrow), widespread abnormal functional connectivity during mixed treatments, and electroconvulsive therapy (ECT)-induced effects specifically on the limbic system (red arrow).

This review indicates that connectivity abnormalities are associated with symptoms in TRS. In particular, the studies noted frontal hypoconnectivity before the initiation of treatment with CLZ or riluzole, an increase in frontal connectivity after riluzole treatment, fronto-temporal hypoconnectivity that may be specific for non-responders, widespread abnormal functional connectivity during mixed treatments, and ECT-induced effects specifically on the limbic system (Figure 3). Functional irregularities in frontal brain regions, especially in the PFC, are consistent with previous SCZ literature and the dysconnectivity hypothesis. Although different treatment methods have the possibility to improve symptoms and abnormal functional connectivity in SCZ, individuals who benefit from each treatment may vary. Whether functional connectivity could help to answer the better treatment method for each individual, remains unknown. However, better definitions of treatment responders and non-responders (according to the TRRIP guidelines) are necessary in future longitudinal studies to correctly differentiate brain regions underlying the pathophysiology of SCZ, which could serve as potential functional biomarkers for treatment resistance. In addition, both the initiation and the duration of TRS should be taken into account. Longitudinal studies with MRI acquisitions both before and after the initiation of different treatments are needed, and the recruitment of additional control cohorts (e.g., NTRS and HC) is necessary. Future studies should be acquired with standardized MRI protocols to facilitate multi-site studies in order to reach larger sample sizes and to improve the reproducibility of the results. Replicability could be further improved with data sharing. The reliability of the rs-fMRI findings could be improved by acquisitions of multiband or multiecho sequences together with fieldmaps. In addition, multimodal MRI studies which consist of structural, diffusion tensor imaging, fMRI, and MR spectroscopy sequences may provide more accurate measures than one modality alone. An interesting future research direction would be to study differences in the effective and dynamic functional connectivity (Ramirez-Mahaluf et al., 2022). In fact, none of the studies reviewed applied these connectivity methods in TRS. In addition, studies which combine MRI information with other clinical and biological parameters could provide important information on the pathophysiology of SCZ and particularly TRS.

## Author contributions

NT designed and conceptualized the review, reviewed and selected the literature, analyzed the results, and wrote the first draft of the manuscript. AH critically revised the review. Both authors contributed to and have approved the final manuscript.

## References

[B1] Ahmed M. Cannon D. M. Scanlon C. Holleran L. Schmidt H. McFarland J.. (2015). Progressive brain atrophy and cortical thinning in schizophrenia after commencing clozapine treatment. NPP 40, 2409–2417. 10.1038/npp.2015.90 25829144 PMC4538355

[B2] Algumaei A. H. Algunaid R. F. Rushdi M. A. Yassine I. A. (2022). Feature and decision-level fusion for schizophrenia detection based on resting-state fMRI data. PLoS ONE 17, e0265300. 10.1371/journal.pone.0265300 35609033 PMC9129055

[B3] Alonso-Solís A. Vives-Gilabert Y. Grasa E. Portella M. J. Rabella M. Sauras R. B.. (2015). Resting-state functional connectivity alterations in the default network of schizophrenia patients with persistent auditory verbal hallucinations. Schizophr. Res. 161, 261–268. 10.1016/j.schres.2014.10.047 25468173

[B4] Andrews-Hanna J. R. Reidler J. S. Sepulcre J. Poulin R. Buckner R. L. (2010). Functional-anatomic fractionation of the brain's default network. Neuron 65, 550–562. 10.1016/j.neuron.2010.02.005 20188659 PMC2848443

[B5] Arango C. Breier A. McMahon R. Carpenter W. T. Jr. Buchanan R. W. (2003). The relationship of clozapine and haloperidol treatment response to prefrontal, hippocampal, and caudate brain volumes. Am. J. Psychiatry 160, 1421–1427. 10.1176/appi.ajp.160.8.1421 12900303

[B6] Blazer A. Chengappa K. N. R. Foran W. Parr A. C. Kahn C. E. Luna B.. (2022). Changes in corticostriatal connectivity and striatal tissue iron associated with efficacy of clozapine for treatment-resistant schizophrenia. Psychopharmacology 239, 2503–2514. 10.1007/s00213-022-06138-0 35435461 PMC9013738

[B7] Buckner R. L. Andrews-Hanna J. R. Schacter D. L. (2008). The brain's default network: anatomy, function, and relevance to disease. Ann. N. Y. Acad. Sci. 1124, 1–38. 10.1196/annals.1440.011 18400922

[B8] Cachia A. Cury C. Brunelin J. Plaze M. Delmaire C. Oppenheim C.. (2020). Deviations in early hippocampus development contribute to visual hallucinations in schizophrenia. Transl. Psychiatry 10, 102. 10.1038/s41398-020-0779-9 32214096 PMC7096500

[B9] Campana M. Falkai P. Siskind D. Hasan A. Wagner E. (2021). Characteristics and definitions of ultra-treatment-resistant schizophrenia - a systematic review and meta-analysis. Schizophr. Res. 228, 218–226. 10.1016/j.schres.2020.12.002 33454644

[B10] Chakrabarti S. (2021). Clozapine resistant schizophrenia: newer avenues of management. World J. Psychiatry 11, 429–448. 10.5498/wjp.v11.i8.429 34513606 PMC8394694

[B11] Chan N. K. Kim J. Shah P. Brown E. E. Plitman E. Carravaggio F.. (2019). Resting-state functional connectivity in treatment response and resistance in schizophrenia: a systematic review. Schizophr. Res. 211, 10–20. 10.1016/j.schres.2019.07.020 31331784

[B12] Chen X. Duan M. He H. Yang M. Klugah-Brown B. Xu H.. (2016). Functional abnormalities of the right posterior insula are related to the altered self-experience in schizophrenia. Psychiatry Res. Neuroimaging 256, 26–32. 10.1016/j.pscychresns.2016.09.006 27662482

[B13] Conley R. R. Kelly D. L. (2001). Management of treatment resistance in schizophrenia. Biol. Psychiatry 50, 898–911. 10.1016/S0006-3223(01)01271-9 11743944

[B14] Dandash O. Fornito A. Lee J. Keefe R. S. Chee M. W. Adcock R. A.. (2014). Altered striatal functional connectivity in subjects with an at-risk mental state for psychosis. Schizophr. Bull. 40, 904–913. 10.1093/schbul/sbt093 23861539 PMC4059431

[B15] Demjaha A. Murray R. M. McGuire P. K. Kapur S. Howes O. D. (2012). Dopamine synthesis capacity in patients with treatment-resistant schizophrenia. Am. J. Psychiatry 169, 1203–1210. 10.1176/appi.ajp.2012.12010144 23034655

[B16] Di Martino A. Scheres A. Margulies D. S. Kelly A. M. Uddin L. Q. Shehzad Z.. (2008). Functional connectivity of human striatum: a resting state FMRI study. Cereb. Cortex 18, 2735–2747. 10.1093/cercor/bhn041 18400794

[B17] Eickhoff S. B. Stephan K. E. Mohlberg H. Grefkes C. Fink G. R. Amunts K.. (2005). A new SPM toolbox for combining probabilistic cytoarchitectonic maps and functional imaging data. Neuroimage 25, 1325–1335. 10.1016/j.neuroimage.2004.12.034 15850749

[B18] Fan L. Li H. Zhuo J. Zhang Y. Wang J. Chen L.. (2016). The human brainnetome atlas: a new brain atlas based on connectional architecture. Cereb. Cortex 26, 3508–3526. 10.1093/cercor/bhw157 27230218 PMC4961028

[B19] Farokhnia M. Sabzabadi M. Pourmahmoud H. Khodaie-Ardakani M. R. Hosseini S. M. Yekehtaz H.. (2014). A double-blind, placebo controlled, randomized trial of riluzole as an adjunct to risperidone for treatment of negative symptoms in patients with chronic schizophrenia. Psychopharmacology 231, 533–542. 10.1007/s00213-013-3261-z 24013610

[B20] Feng S. Zheng S. Zou H. Dong L. Zhu H. Liu S.. (2022). Altered functional connectivity of cerebellar networks in first-episode schizophrenia. Front. Cell Neurosci. 16, 1024192. 10.3389/fncel.2022.1024192 36439199 PMC9692071

[B21] Fornito A. Zalesky A. Pantelis C. Bullmore E. T. (2012). Schizophrenia, neuroimaging and connectomics. Neuroimage 62, 2296–2314. 10.1016/j.neuroimage.2011.12.090 22387165

[B22] Friston K. Brown H. R. Siemerkus J. Stephan K. E. (2016). The dysconnection hypothesis 2016. Schizophr. Res. 176, 83–94. 10.1016/j.schres.2016.07.014 27450778 PMC5147460

[B23] Ganella E. P. Bartholomeusz C. F. Seguin C. Whittle S. Bousman C. Phassouliotis C.. (2017). Functional brain networks in treatment-resistant schizophrenia. Schizophr. Res. 184, 73–81. 10.1016/j.schres.2016.12.008 28011131

[B24] Ganella E. P. Seguin C. Bartholomeusz C. F. Whittle S. Bousman C. Wannan C. M. J.. (2018). Risk and resilience brain networks in treatment-resistant schizophrenia. Schizophr Res. 193, 284–292. 10.1016/j.schres.2017.07.014 28735641

[B25] Gao S. Lu S. Shi X. Ming Y. Xiao C. Sun J.. (2018). Distinguishing between treatment-resistant and non-treatment-resistant schizophrenia using regional homogeneity. Front. Psychiatry 9, 282. 10.3389/fpsyt.2018.00282 30127752 PMC6088138

[B26] Goldstein M. E. Anderson V. M. Pillai A. Kydd R. R. Russell B. R. (2015). Glutamatergic neurometabolites in clozapine-responsive and -resistant schizophrenia. Int. J. Neuropsychopharmacol. 18, pyu117. 10.1093/ijnp/pyu117 25603859 PMC4438552

[B27] Gong J. Cui L. B. Xi Y. B. Zhao Y. S. Yang X. J. Xu Z. L.. (2020). Predicting response to electroconvulsive therapy combined with antipsychotics in schizophrenia using multi-parametric magnetic resonance imaging. Schizophr. Res. 216, 262–271. 10.1016/j.schres.2019.11.046 31826827

[B28] Groppe D. M. Urbach T. P. Kutas M. (2011). Mass univariate analysis of event-related brain potentials/fields I: a critical tutorial review. Psychophysiology 48, 1711–1725. 10.1111/j.1469-8986.2011.01273.x 21895683 PMC4060794

[B29] Grover S. Sarkar S. Sahoo S. (2022). Augmentation strategies for clozapine resistance: A systematic review and meta-analysis. Acta Neuropsychiatr. 35, 65–75. 10.1017/neu.2022.30 36380513

[B30] Hadley J. A. Kraguljac N. V. White D. M. Ver Hoef L. Tabora J. Lahti A. C.. (2016). Change in brain network topology as a function of treatment response in schizophrenia: a longitudinal resting-state fMRI study using graph theory. NPJ Schizophr. 2, 16014. 10.1038/npjschz.2016.14 27336056 PMC4898893

[B31] Hall R. C. Parks J. (1995). The modified global assessment of functioning scale: addendum. Psychosomatics 36, 416–417. 10.1016/S0033-3182(95)71656-5 7652146

[B32] Hedlund J. Vieweg B. (1980). The brief psychiatric rating scale (BPRS): a comprehensive review. J. Oper. Psychiatry 11, 48–65.

[B33] Howes O. McCutcheon R. Stone J. (2015). Glutamate and dopamine in schizophrenia: an update for the 21st century. J. Psychopharmacol. 29, 97–115. 10.1177/0269881114563634 25586400 PMC4902122

[B34] Howes O. D. McCutcheon R. Agid O. de Bartolomeis A. van Beveren N. J. Birnbaum M. L.. (2017). Treatment-resistant schizophrenia: treatment response and resistance in psychosis (TRRIP) working group consensus guidelines on diagnosis and terminology. Am. J. Psychiatry 174, 216–229. 10.1176/appi.ajp.2016.16050503 27919182 PMC6231547

[B35] Hu H. Jiang Y. Xia M. Tang Y. Zhang T. Cui H.. (2022). Functional reconfiguration of cerebellum-cerebral neural loop in schizophrenia following electroconvulsive therapy. Psychiatry Res. Neuroimaging 320, 111441. 10.1016/j.pscychresns.2022.111441 35085957

[B36] Hu M. L. Zong X. F. Mann J. J. Zheng J. J. Liao Y. H. Li Z. C.. (2017). A review of the functional and anatomical default mode network in schizophrenia. Neurosci. Bull. 33, 73–84. 10.1007/s12264-016-0090-1 27995564 PMC5567552

[B37] Hu Q. Huang H. Jiang Y. Jiao X. Zhou J. Tang Y.. (2022). Temporoparietal connectivity within default mode network associates with clinical improvements in schizophrenia following modified electroconvulsive therapy. Front. Psychiatry 12, 768279. 10.3389/fpsyt.2021.768279 35058815 PMC8763790

[B38] Huang H. Jiang Y. Xia M. Tang Y. Zhang T. Cui H.. (2018). Increased resting-state global functional connectivity density of default mode network in schizophrenia subjects treated with electroconvulsive therapy. Schizophr. Res. 197, 192–199. 10.1016/j.schres.2017.10.044 29117910

[B39] Hwang K. Bertolero M. A. Liu W. B. D'Esposito M. (2017). The human thalamus is an integrative hub for functional brain networks. J. Neurosci. 37, 5594–5607. 10.1523/JNEUROSCI.0067-17.2017 28450543 PMC5469300

[B40] Iwata Y. Nakajima S. Plitman E. Caravaggio F. Kim J. Shah P.. (2019). Glutamatergic neurometabolite levels in patients with ultra-treatment-resistant schizophrenia: a cross-sectional 3T proton magnetic resonance spectroscopy study. Biol. Psychiatry 85, 596–605. 10.1016/j.biopsych.2018.09.009 30389132

[B41] Jiang L. Zuo X. N. (2016). Regional homogeneity: a multimodal, multiscale neuroimaging marker of the human connectome. Neuroscientist 22, 486–505. 10.1177/1073858415595004 26170004 PMC5021216

[B42] Jiang Y. Patton M. H. Zakharenko S. S. A. (2021). Case for thalamic mechanisms of schizophrenia: perspective from modeling 22q11.2 deletion syndrome. Front. Neural Circuits 15, 769969. 10.3389/fncir.2021.769969 34955759 PMC8693383

[B43] Jiang Y. Xia M. Li X. Tang Y. Li C. Huang H.. (2019a). Insular changes induced by electroconvulsive therapy response to symptom improvements in schizophrenia. Prog. Neuropsychopharmacol. Biol. Psychiatry 89, 254–262. 10.1016/j.pnpbp.2018.09.009 30248379

[B44] Jiang Y. Xu L. Li X. Tang Y. Wang P. Li C.. (2019b). Common increased hippocampal volume but specific changes in functional connectivity in schizophrenia patients in remission and non-remission following electroconvulsive therapy: a preliminary study. Neuroimage Clin. 24, 102081. 10.1016/j.nicl.2019.102081 31734526 PMC6861644

[B45] Kane J. Honigfeld G. Singer J. Meltzer H. (1988). Clozapine for the treatment-resistant schizophrenic. A double-blind comparison with chlorpromazine. Arch. Gen. Psychiatry 45, 789–796. 10.1001/archpsyc.1988.01800330013001 3046553

[B46] Kane J. M. Agid O. Baldwin M. L. Howes O. Lindenmayer J. P. Marder S.. (2019). Clinical guidance on the identification and management of treatment-resistant schizophrenia. J. Clin. Psychiatry 80, 18co.m12123. 10.4088/JCP.18com12123 30840788

[B47] Kay S. R. Fiszbein A. Lewis A. (1987). The Positive and Negative Syndrome Scale (PANSS) for schizophrenia. Schizophr. Bull. 13, 261–276. 10.1093/schbul/13.2.261 3616518

[B48] Kim W. S. Shen J. Tsogt U. Odkhuu S. Chung Y. C. (2022). Altered thalamic subregion functional networks in patients with treatment-resistant schizophrenia. World J. Psychiatry 12, 693–707. 10.5498/wjp.v12.i5.693 35663295 PMC9150031

[B49] Kochunov P. Huang J. Chen S. Li Y. Tan S. Fan F.. (2019). White matter in schizophrenia treatment resistance. Am. J. Psychiatry 176, 829–838. 10.1176/appi.ajp.2019.18101212 31352812 PMC6773514

[B50] Krajner F. Hadaya L. McQueen G. Sendt K. V. Gillespie A. Avila A.. (2022). Subcortical volume reduction and cortical thinning 3 months after switching to clozapine in treatment resistant schizophrenia. Schizophrenia 8, 13. 10.1038/s41537-022-00230-2 35236831 PMC8891256

[B51] Lawrie S. M. Buechel C. Whalley H. C. Frith C. D. Friston K. J. Johnstone E. C.. (2002). Reduced frontotemporal functional connectivity in schizophrenia associated with auditory hallucinations. Biol. Psychiatry 51, 1008–1011. 10.1016/S0006-3223(02)01316-1 12062886

[B52] Leaver A. M. Espinoza R. Wade B. Narr K. L. (2022). Parsing the network mechanisms of electroconvulsive therapy. Biol. Psychiatry 92, 193–203. 10.1016/j.biopsych.2021.11.016 35120710 PMC9196257

[B53] Leaver A. M. Wade B. Vasavada M. Hellemann G. Joshi S. H. Espinoza R.. (2018). Fronto-temporal connectivity predicts ECT outcome in major depression. Front. Psychiatr 9, 92. 10.3389/fpsyt.2018.00092 29618992 PMC5871748

[B54] Leroux E. Vandevelde A. Tréhout M. Dollfus S. (2018). Abnormalities of fronto-subcortical pathways in schizophrenia and the differential impacts of antipsychotic treatment: a DTI-based tractography study. Psychiatry Res. Neuroimaging 280, 22–29. 10.1016/j.pscychresns.2018.08.008 30145382

[B55] Liao W. Zhang Z. Pan Z. Mantini D. Ding J. Duan X.. (2011). Default mode network abnormalities in mesial temporal lobe epilepsy: a study combining fMRI and DTI. Hum. Brain Mapp. 32, 883–895. 10.1002/hbm.21076 20533558 PMC6870458

[B56] Liu C. Kim W. S. Shen J. Tsogt U. Kang N. I. Lee K. H.. (2022). Altered neuroanatomical signatures of patients with treatment-resistant schizophrenia compared to patients with early-stage schizophrenia and healthy controls. Front. Psychiatry 13, 802025. 10.3389/fpsyt.2022.802025 35664476 PMC9158464

[B57] Luo C. Lencer R. Hu N. Xiao Y. Zhang W. Li S.. (2020). Characteristics of white matter structural networks in chronic schizophrenia treated with clozapine or risperidone and those never treated. Int. J. Neuropsychopharmacol. 23, 799–810. 10.1093/ijnp/pyaa061 32808036 PMC7770521

[B58] Luo C. Li Q. Lai Y. Xia Y. Qin Y. Liao W.. (2011). Altered functional connectivity in default mode network in absence epilepsy: a resting-state fMRI study. Hum. Brain Mapp. 32, 438–449. 10.1002/hbm.21034 21319269 PMC6870112

[B59] Lynch C. J. Power J. D. Scult M. A. Dubin M. Gunning F. M. Liston C.. (2020). Rapid precision functional mapping of individuals using multi-echo fMRI. Cell Rep. 33, 108540. 10.1016/j.celrep.2020.108540 33357444 PMC7792478

[B60] Margulies D. S. Kelly A. M. Uddin L. Q. Biswal B. B. Castellanos F. X. Milham M. P.. (2007). Mapping the functional connectivity of anterior cingulate cortex. Neuroimage 37, 579–588. 10.1016/j.neuroimage.2007.05.019 17604651

[B61] McCutcheon R. A. Abi-Dargham A. Howes O. D. (2019). Schizophrenia, dopamine and the striatum: from biology to symptoms. Trends Neurosci. 42, 205–220. 10.1016/j.tins.2018.12.004 30621912 PMC6401206

[B62] McNabb C. B. Kydd R. Sundram F. Soosay I. Russell B. R. (2018c). Differences in white matter connectivity between treatment-resistant and treatment-responsive subtypes of schizophrenia. Psychiatry Res. Neuroimaging 282, 47–54. 10.1016/j.pscychresns.2018.11.002 30412902

[B63] McNabb C. B. Sundram F. Soosay I. Kydd R. R. Russell B. R. (2018a). Increased sensorimotor network connectivity associated with clozapine eligibility in people with schizophrenia. Psychiatry Res. Neuroimaging 275, 36–42. 10.1016/j.pscychresns.2018.02.008 29650266

[B64] McNabb C. B. Tait R. J. McIlwain M. E. Anderson V. M. Suckling J. Kydd R. R.. (2018b). Functional network dysconnectivity as a biomarker of treatment resistance in schizophrenia. Schizophr. Res. 195, 160–167. 10.1016/j.schres.2017.10.015 29042073

[B65] McQueen G. Sendt K. V. Gillespie A. Avila A. Lally J. Vallianatou K.. (2021). Changes in brain glutamate on switching to clozapine in treatment-resistant schizophrenia. Schizophr. Bull. 47, 662–671. 10.1093/schbul/sbaa156 33398325 PMC8084451

[B66] Molent C. Olivo D. Wolf R. C. Balestrieri M. Sambataro F. (2019). Functional neuroimaging in treatment resistant schizophrenia: a systematic review. Neurosci. Biobehav. Rev. 104, 178–190. 10.1016/j.neubiorev.2019.07.001 31276716

[B67] Molina V. Reig S. Sarramea F. Sanz J. Francisco Artaloytia J. Luque R.. (2003). Anatomical and functional brain variables associated with clozapine response in treatment-resistant schizophrenia. Psychiatry Res. 124, 153–161. 10.1016/S0925-4927(03)00108-2 14623067

[B68] Molina V. Taboada D. Aragüés M. Hernández J. A. Sanz-Fuentenebro J. (2014). Greater clinical and cognitive improvement with clozapine and risperidone associated with a thinner cortex at baseline in first-episode schizophrenia. Schizophr. Res. 158, 223–229. 10.1016/j.schres.2014.06.042 25088730

[B69] Moon S. Y. Kim M. Lho S. K. Oh S. Kim S. H. Kwon J. S.. (2021). Systematic review of the neural effect of electroconvulsive therapy in patients with schizophrenia: hippocampus and insula as the key regions of modulation. Psychiatry Investig. 18, 486–499. 10.30773/pi.2020.0438 34218638 PMC8256139

[B70] Mouaffak F. Tranulis C. Gourevitch R. Poirier M. F. Douki S. Olié J. P.. (2006). Augmentation strategies of clozapine with antipsychotics in the treatment of ultraresistant schizophrenia. Clin. Neuropharmacol. 29, 28–33. 10.1097/00002826-200601000-00009 16518132

[B71] Mouchlianitis E. Bloomfield M. A. Law V. Beck K. Selvaraj S. Rasquinha N.. (2016). Treatment-resistant schizophrenia patients show elevated anterior cingulate cortex glutamate compared to treatment-responsive. Schizophr. Bull. 42, 744–752. 10.1093/schbul/sbv151 26683625 PMC4838083

[B72] Mwansisya T. E. Hu A. Li Y. Chen X. Wu G. Huang X.. (2017). Task and resting-state fMRI studies in first-episode schizophrenia: a systematic review. Schizophr. Res. 189, 9–18. 10.1016/j.schres.2017.02.026 28268041

[B73] Ochi R. Noda Y. Tsuchimoto S. Tarumi R. Honda S. Matsushita K.. (2020). White matter microstructural organizations in patients with severe treatment-resistant schizophrenia: a diffusion tensor imaging study. Prog. Neuropsychopharmacol. Biol. Psychiatry 100, 109871. 10.1016/j.pnpbp.2020.109871 31962187

[B74] Ozcelik-Eroglu E. Ertugrul A. Oguz K. K. Has A. C. Karahan S. Yazici M. K.. (2014). Effect of clozapine on white matter integrity in patients with schizophrenia: a diffusion tensor imaging study. Psychiatry Res. 223, 226–235. 10.1016/j.pscychresns.2014.06.001 25012780

[B75] Pergola G. Selvaggi P. Trizio S. Bertolino A. Blasi G. (2015). The role of the thalamus in schizophrenia from a neuroimaging perspective. Neurosci. Biobehav. Rev. 54, 57–75. 10.1016/j.neubiorev.2015.01.013 25616183

[B76] Pillinger T. Rogdaki M. McCutcheon R. A. Hathway P. Egerton A. Howes O. D.. (2019). Altered glutamatergic response and functional connectivity in treatment resistant schizophrenia: the effect of riluzole and therapeutic implications. Psychopharmacology 236, 1985–1997. 10.1007/s00213-019-5188-5 30820633 PMC6642056

[B77] Ramirez-Mahaluf J. P. Tepper Á. Alliende L. M. Mena C. Castañeda C. P. Iruretagoyena B.. (2022). Dysconnectivity in schizophrenia revisited: abnormal temporal organization of dynamic functional connectivity in patients with a first episode of psychosis. Schizophr. Bull. sbac187. 10.1093/schbul/sbac187. [Epub ahead of print]. 36472382 PMC10154721

[B78] Rubinov M. Bullmore E. (2013). Schizophrenia and abnormal brain network hubs. Dialogues Clin. Neurosci. 15, 339–349. 10.31887/DCNS.2013.15.3/mrubinov 24174905 PMC3811105

[B79] Rubinov M. Sporns O. (2010). Complex network measures of brain connectivity: uses and interpretations. Neuroimage 52, 1059–1069. 10.1016/j.neuroimage.2009.10.003 19819337

[B80] Sarpal D. K. Blazer A. Wilson J. D. Calabro F. J. Foran W. Kahn C. E.. (2022a). Relationship between plasma clozapine/N-desmethylclozapine and changes in basal forebrain-dorsolateral prefrontal cortex coupling in treatment-resistant schizophrenia. Schizophr. Res. 243, 170–177. 10.1016/j.schres.2022.03.014 35381515 PMC9189030

[B81] Sarpal D. K. Tarcijonas G. Calabro F. J. Foran W. Haas G. L. Luna B.. (2022b). Context-specific abnormalities of the central executive network in first-episode psychosis: relationship with cognition. Psychol. Med. 52, 2299–2308. 10.1017/S0033291720004201 33222723 PMC9805803

[B82] Scheepers F. E. de Wied C. C. Hulshoff Pol H. E. van de Flier W. van der Linden J. A. Kahn R. S.. (2001). The effect of clozapine on caudate nucleus volume in schizophrenic patients previously treated with typical antipsychotics. NPP 24, 47–54. 10.1016/S0893-133X(00)00172-X 11106875

[B83] Schmidt M. F. (1996). Rey Auditory and Verbal Learning Test: A Handbook. Los Angeles, CA: Western Psychological Services.

[B84] Shah P. Plitman E. Iwata Y. Kim J. Nakajima S. Chan N.. (2020). Glutamatergic neurometabolites and cortical thickness in treatment-resistant schizophrenia: implications for glutamate-mediated excitotoxicity. J. Psychiatr. Res. 124, 151–158. 10.1016/j.jpsychires.2020.02.032 32169688

[B85] Siskind D. Siskind V. Kisely S. (2017). Clozapine response rates among people with treatment-resistant schizophrenia: data from a systematic review and meta-analysis. Can. J. Psychiatry 62, 772–777. 10.1177/0706743717718167 28655284 PMC5697625

[B86] Smart S. E. Kepińska A. P. Murray R. M. MacCabe J. H. (2021). Predictors of treatment resistant schizophrenia: a systematic review of prospective observational studies. Psycho. Med. 51, 44–53. 10.1017/S0033291719002083 31462334 PMC7856410

[B87] Sporns O. (2013). Network attributes for segregation and integration in the human brain. Curr. Opin. Neurobiol. 23, 162–171. 10.1016/j.conb.2012.11.015 23294553

[B88] Stephan K. E. Friston K. J. Frith C. D. (2009). Dysconnection in schizophrenia: from abnormal synaptic plasticity to failures of self-monitoring. Schizophr. Bull. 35, 509–527. 10.1093/schbul/sbn176 19155345 PMC2669579

[B89] Suzuki T. Remington G. Mulsant B. H. Uchida H. Rajji T. K. Graff-Guerrero A.. (2012). Defining treatment-resistant schizophrenia and response to antipsychotics: a review and recommendation. Psychiatry Res. 197, 1–6. 10.1016/j.psychres.2012.02.013 22429484

[B90] Tarumi R. Tsugawa S. Noda Y. Plitman E. Honda S. Matsushita K.. (2020). Levels of glutamatergic neurometabolites in patients with severe treatment-resistant schizophrenia: a proton magnetic resonance spectroscopy study. NPP 45, 632–640. 10.1038/s41386-019-0589-z 31842203 PMC7021829

[B91] Thomann P. A. Wolf R. C. Nolte H. M. Hirjak D. Hofer S. Seidl U.. (2017). Neuromodulation in response to electroconvulsive therapy in schizophrenia and major depression. Brain Stimul. 10, 637–644. 10.1016/j.brs.2017.01.578 28162976

[B92] Tronchin G. Akudjedu T. N. Ahmed M. Holleran L. Hallahan B. Cannon D. M.. (2020). Progressive subcortical volume loss in treatment-resistant schizophrenia patients after commencing treatment with clozapine. NPP 45, 1353–1361. 10.1038/s41386-020-0665-4 32268345 PMC7298040

[B93] Tronchin G. McPhilemy G. Ahmed M. Kilmartin L. Costello L. Forde N. J.. (2021). White matter microstructure and strutural networks in treatment-resistant schizophrenia patients after commencing clozapine treatment: a longitudinal diffusion imaging study. Psychiatry Res. 298, 113772. 10.1016/j.psychres.2021.113772 33556689

[B94] Tzourio-Mazoyer N. Landeau B. Papathanassiou D. Crivello F. Etard O. Delcroix N.. (2002). Automated anatomical labeling of activations in SPM using a macroscopic anatomical parcellation of the MNI MRI single-subject brain. Neuroimage 15, 273–289. 10.1006/nimg.2001.0978 11771995

[B95] Wang J. Jiang Y. Tang Y. Xia M. Curtin A. Li J.. (2020). Altered functional connectivity of the thalamus induced by modified electroconvulsive therapy for schizophrenia. Schizophr. Res. 218, 209–218. 10.1016/j.schres.2019.12.044 31956007

[B96] Wang J. Tang Y. Curtin A. Xia M. Tang X. Zhao Y.. (2019). ECT-induced brain plasticity correlates with positive symptom improvement in schizophrenia by voxel-based morphometry analysis of grey matter. Brain Stimul. 12, 319–328. 10.1016/j.brs.2018.11.006 30473477

[B97] White T. P. Wigton R. Joyce D. W. Collier T. Fornito A. Shergill S. S.. (2016). Dysfunctional striatal systems in treatment-resistant schizophrenia. Neuropsychopharmacology 41, 1274–1285. 10.1038/npp.2015.277 26346637 PMC4793111

[B98] Yang X. Xu Z. Xi Y. Sun J. Liu P. Liu P.. (2020). Predicting responses to electroconvulsive therapy in schizophrenia patients undergoing antipsychotic treatment: baseline functional connectivity among regions with strong electric field distributions. Psychiatry Res. Neuroimaging 299, 111059. 10.1016/j.pscychresns.2020.111059 32135406

[B99] Yoshimura B. Yada Y. So R. Takaki M. Yamada N. (2017). The critical treatment window of clozapine in treatment-resistant schizophrenia: secondary analysis of an observational study. Psychiatry Res. 250, 65–70. 10.1016/j.psychres.2017.01.064 28142068

[B100] Zaborszky L. Hoemke L. Mohlberg H. Schleicher A. Amunts K. Zilles K.. (2008). Stereotaxic probabilistic maps of the magnocellular cell groups in human basal forebrain. Neuroimage 42, 1127–1141. 10.1016/j.neuroimage.2008.05.055 18585468 PMC2577158

[B101] Zhou Y. Fan L. Qiu C. Jiang T. (2015). Prefrontal cortex and the dysconnectivity hypothesis of schizophrenia. Neurosci Bull. 31, 207–219. 10.1007/s12264-014-1502-8 25761914 PMC5563697

[B102] Zugman A. Gadelha A. Assunção I. Sato J. Ota V. K. Rocha D. L.. (2013). Reduced dorso-lateral prefrontal cortex in treatment resistant schizophrenia. Schizophr. Res. 148, 81–86. 10.1016/j.schres.2013.05.002 23721966

